# Crime in Philadelphia: Bayesian Clustering with Particle Optimization

**DOI:** 10.1080/01621459.2022.2156348

**Published:** 2023-01-18

**Authors:** Cecilia Balocchi, Sameer K. Deshpande, Edward I. George, Shane T. Jensen

**Affiliations:** a School of Mathematics, University of Edinburgh, Edinburgh, UK; b Department of Statistics, University of Wisconsin–Madison, Madison, WI; c Department of Statistics, University of Pennsylvania, Philadelphia, PA

**Keywords:** Bayesian model averaging, Boundary detection, Spatial clustering, Spatial smoothness, Urban analytics, Variational inference

## Abstract

Accurate estimation of the change in crime over time is a critical first step toward better understanding of public safety in large urban environments. Bayesian hierarchical modeling is a natural way to study spatial variation in urban crime dynamics at the neighborhood level, since it facilitates principled “sharing of information” between spatially adjacent neighborhoods. Typically, however, cities contain many physical and social boundaries that may manifest as spatial discontinuities in crime patterns. In this situation, standard prior choices often yield overly smooth parameter estimates, which can ultimately produce mis-calibrated forecasts. To prevent potential over-smoothing, we introduce a prior that partitions the set of neighborhoods into several clusters and encourages spatial smoothness within each cluster. In terms of model implementation, conventional stochastic search techniques are computationally prohibitive, as they must traverse a combinatorially vast space of partitions. We introduce an ensemble optimization procedure that simultaneously identifies several high probability partitions by solving one optimization problem using a new local search strategy. We then use the identified partitions to estimate crime trends in Philadelphia between 2006 and 2017. On simulated and real data, our proposed method demonstrates good estimation and partition selection performance. Supplementary materials for this article are available online.

## 1 Introduction

Beginning in the late 2000s and continuing through the 2010s, Philadelphia experienced population growth, underwent a rapid evolution in its built environment, and observed a generally decreasing trend in the total number of violent crimes reported in the city. Although the *total* number of violent crimes decreased city-wide, the decreasing trend was not experienced uniformly in all of the local neighborhoods making up the city. In this article, we carefully estimate both the neighborhood-level baseline levels and temporal trends in crime and separately cluster these estimates. This enables us to identify clusters of neighborhoods whose trends in crime deviated markedly from the overall city-wide decrease as well as clusters of neighborhoods displaying substantially higher or lower baseline levels than surrounding neighborhoods.

Accurate modeling of the neighborhood-level crime patterns benefits many stakeholders: urban planners can better understand how socioeconomic factors and the built environment affect crime, city officials can develop community programs and interventions to improve the overall quality of life for all residents, and law enforcement can more thoughtfully deploy resources to increase public safety. Accurate modeling of neighborhood trends is, however, complicated by several factors. First, simple comparisons of the temporal trajectories of neighborhood-level counts fail to account for important differences in size, land use and zoning, population, and the socio-demographic makeup of each neighborhood. Consequently, in order to facilitate accurate comparisons across neighborhoods, we directly model the *crime density*—defined as the number of violent crimes divided by the land area (in square miles)—of each neighborhood over time. As we detail in Section 2, area is a more principled normalization than population for small urban areas.

The second complication lies in the spatial modeling of crime density. Bayesian hierarchical modeling is an attractive way to study crime at the neighborhood level as it allows us to “borrow strength” between spatially adjacent neighborhoods. Recently, Balocchi and Jensen (2019) demonstrated that Bayesian models that encourage spatial smoothing between neighborhoods with conditionally autoregressive (CAR; Besag 1974) priors yield more accurate neighborhood-level forecasts than fitting independent models to each neighborhood. Though CAR models are an intuitive and popular way to introduce spatial dependence, they can produce overly smooth parameter estimates and forecasts that are at odds with the realities of complex urban environments. In fact, as we will see in Section 2, while crime in Philadelphia displays considerable spatial correlation, there are also sharp discontinuities. While some discontinuities coincide with known landmarks like major streets, parks, and bodies of water, several occur along less obvious boundaries that may result from differences in socioeconomic conditions and disparate effects of public policy initiatives like community crime watches.

Specifying a CAR prior without accounting for potential discontinuities can lead to poor estimation of crime around these geographic or socioeconomic barriers. Directly modeling the locations of individual discontinuities between pairs of neighborhoods typically relies on heavily over-parameterized models; in fact, many such models introduce one latent parameter for each pair of adjacent neighborhoods. We instead aim to identify *clusters* of neighborhoods with similar crime dynamics.

In this article, we propose a “CAR–within–clusters” model where we introduce *two latent spatial partitions* of neighborhoods in Philadelphia, one for the baseline levels of crime and one for the time trends. We then specify separate CAR priors on the neighborhood-specific parameters within each cluster of each partition. Unlike many classical approaches to Bayesian spatial clustering, our proposed model allows (i) the spatial distribution of the baseline levels of crime to differ from that of the time trends and (ii) the parameters to vary both within and between clusters.

Because we allow different model parameters to cluster differently, the combinatorial vastness of the underlying product space of partitions renders stochastic search techniques like Markov chain Monte Carlo (MCMC) computationally prohibitive. We instead focus on posterior optimization. However, rather than simply finding the *maximum a posteriori* (MAP) partitions, we propose an extension of Ročková (2018)’s ensemble optimization framework that simultaneously identifies multiple partitions with high posterior probability by solving a *single* optimization problem. Solving this problem is formally equivalent to finding a variational approximation of the discrete posterior distribution over the partitions. We introduce a new local search strategy that, at a high level, runs several greedy searches that are made “mutually aware” through an entropy penalty. This penalty promotes diversity among the estimated partitions by discouraging different search paths from visiting the same point in the latent discrete space.

Here is an outline for the rest of the article. In Section 2 we describe our crime data and introduce our “CAR–within–clusters” model. Then, in Section 2.1, we highlight important similarities and differences between our proposed model and existing Bayesian spatial clustering methods. We introduce our variational approximation and local optimization strategy in Section 3 before demonstrating its use on synthetic data in Section 4. We then apply our method to the Philadelphia crime data in Section 5. Section 6 concludes with a discussion of our results and an outline of potential future directions. An R package implementing our method and all code and data to replicate the results in this article are available at https://github.com/cecilia-balocchi/particle-optimization.

## 2 Data and the “CAR–within–Clusters” Model

Our crime data comes from opendataphilly.org, where the Philadelphia Police Department publicly releases the location, time, and type of each reported crime in the city. We focus on *violent* crimes, which include homicides, rapes, robberies, and aggravated assaults (FBI 2011), aggregated at the census tract level. Philadelphia is divided into *N* = 384 census tracts, which range in size from 0.04 to 6.65 square miles and whose populations range from 0 to 8300, with mean of 4000, according to the 2010 Census.

When comparing crime incidence across heterogeneous regions, it is very common for criminologists to work with per-capita crime rates (e.g., crime rates per 100,000 inhabitants). However, for small geographic regions within urban environments, Zhang and Peterson (2007) point out that frequently neither criminals nor victims are from the same neighborhood as the crime location. They instead recommend normalizing crime counts by the area of the neighborhood rather than the population.

To this end, let $c_{i,t}$ be the total number of violent crimes reported in neighborhood *i* in year *t*, with *t* = 0 corresponding to the year 2006 and *t* = 11 corresponding to the year 2017. Additionally, let *A_i_* be the area of neighborhood *i* in square miles. Since the distribution of *crime density* $c_{i,t}/A_{i}$ is quite skewed, we transform the densities using the inverse hyperbolic sine transformation: $y_{i,t}=\;\text{log}\;\left(c_{i,t}/A_{i}+(1+{\left(c_{i,t}/A_{i}\right)}^{2}){)}^{1/2}\right)-\;\text{log}\;(2).$ This transformation behaves quite similarly to the logarithmic transform but is well-defined at zero (Burbidge, Magee, and Robb 1988).

At a high-level, we could model, independently for each census tract i=1,…,N and $t=0,\ldots ,11,y_{i,t}∼N(f_{i,0}(x_{t}),\sigma^{2}),$ where *x_t_* is the time index standardized to have mean zero and unit variance and the unknown regression function $f_{i,0}$ measures the expected transformed crime density in neighborhood *i*. Rather than attempt to estimate each $f_{i,0}$ exactly—a task made complicated by the fact that we only have 12 observations per census tract—we instead focus on *approximating* $f_{i,0}$ up to the first order. That is, we introduce parameters *α_i_* and *β_i_* for each census tract and fit the approximate model

(1) $$y_{i,t}∼N(\alpha_{i}+\beta_{i}x_{t},\sigma^{2}).$$

The parameters *α_i_* and *β_i_*, respectively, approximate the mean level and time trend of the transformed crime density in census tract *i*. Although the approximation in (1) may not perfectly characterize the temporal evolution of crime, such linear approximations are routinely used in limited data settings like ours, which includes only 12 observations per census tract (Bernardinelli et al. 1995; Anderson, Lee, and Dean 2017). Moreover, an exploratory analysis (see Section S4.1 in the supplementary materials) did not reveal strong suggestions of nonlinearity in the $y_{i,t}.$ While such a first order approximation is justified in our setting, we note that the methods we develop in Section 3 to estimate and cluster the *α_i_*’s and *β_i_*’s may be extended to higher-order approximations of the $f_{0,i}$’s that can capture non-linearities; we defer discussion of such extensions of Section S4.2 in the supplementary materials.

Although the raw crime counts $c_{i,t}$ were not of primary interest in our analysis, we can, nevertheless, elaborate the model in (1) to directly model the observed crime counts. For instance, following Kowal and Canale (2020), we can model crime counts as a suitably rounded and transformed latent Gaussian random variable. Specifically, we can model $c_{i,t}=bA_{i}\text{sinh}(\;\text{log}\;(2)+y_{\text{it}}^{⋆})e$ where bue is the nearest integer to *u* and $y_{i,t}^{⋆}∼N(\alpha_{i}+\beta_{i}x_{t},\sigma^{2})$ is a latent variable. Recent work (Kowal and Canale 2020; Kowal and Wu 2021) demonstrates that such transformed and truncated latent Gaussian models are more accurate and flexible than conventional Poisson and negative binomial models of counts.

### 2.1 Related Work

The model in (1) is a linear model with spatially varying coefficients. Geographically weighted regression (GWR; Fotheringham, Brunsdon, and Charlton 2002) is a popular method that estimates the parameters $\left(\alpha_{i},\beta_{i}\right)$ in region *i* using data from region *i* and weighted data from nearby regions, with regions closer to *i* receiving higher weights. Within the Bayesian literature, Gaussian processes (GPs) are a common prior choice to induce spatial dependence between the parameters estimated at different locations. In the presence of a priori unknown spatial discontinuities, however, both GWR and GP-based methods can introduce an inappropriate amount of smoothness, resulting in substantial bias in the parameter estimates and downstream crime forecasts.

There is a vast literature on data-adaptive strategies for detecting such discontinuities. One approach involves first fitting a simple model that does not account for potential discontinuities and then identifying jumps in the fitted values (see, e.g., Boots (2001), Li, Banerjee, and McBean (2011), Banerjee et al. (2012), Lu and Carlin (2005), and Lee and Mitchell (2013)). Alternatively, many authors directly model uncertainty about which borders correspond to sharp discontinuities within larger Bayesian hierarchical models (see, e.g., Lu et al. 2007; Lee and Mitchell 2012; Balocchi and Jensen 2019). Despite their intuitive appeal, these latter models are heavily over-parameterized, often introducing one latent parameter for every pair of adjacent neighborhoods.

Rather than looking for individual discontinuities between pairs of neighborhoods, several authors instead look for clusters of neighborhoods with similar parameter values. Such clustered models are much more parsimonious and readily interpreted than models of pairwise discontinuities. Our goal in this work is to cluster and estimate the mean-levels *α_i_* and time-trends *β_i_* in Equation (1). Because there is no a priori reason to expect the spatial distribution of the *α_i_*’s to match the spatial distribution of the *β_i_*’s, we require a method that can estimate separate clusters for both sets of parameters. We further require a method that can recover *spatial partitions*, whose clusters consist of neighborhoods which are spatially adjacent. Unfortunately, most existing methods for spatially clustered regression fail to meet both criteria.

Two important early works on Bayesian spatial clustering are Knorr-Held and Raßer (2000) and Denison and Holmes (2001). Both fit intercept-only Poisson regression models in which the intercepts are constant within clusters. In both methods, clusters are defined with respect to carefully chosen distance metrics. Gangnon and Clayton (2000) and Green and Richardson (2002) also fit Poisson regression models with clustered intercepts. They also assumed that parameters were constant within clusters but specified priors on the partitions that, respectively, depended on the cluster’s shape and number of adjacent units within each cluster. In general, neither of their procedures recover spatial partitions.

Feng et al. (2016) substantially expand Knorr-Held and Raßer’s (2000) model by incorporating additional covariates with spatially clustered effects. Despite producing spatial clusters, their extension assumes that (i) covariate effects are constant within clusters and (ii) the effects of every covariate are clustered identically. Page and Quintana’s (2016) spatial product partition model makes the same assumptions but is further limited by the fact that it places positive prior probability on nonspatial partitions.

Anderson, Lee, and Dean (2017) study the spatiotemporal variation in respiratory disease risk by fitting a Poisson regression model in each areal unit. Like us, their goal is to estimate an intercept and time-trend within each areal unit and, unlike Feng et al. (2016), they do not assume that the intercepts and time-trends are clustered identically. However, they do not restrict attention to spatial clusters and their computational procedure requires the user to specify, a priori, the maximum number of clusters for both the intercept and time-trend.

Recently Teixeira, Assunção, and Loschi (2019) and Li and Sang (2019) proposed elegant methods for spatial clustering based on spanning tree representations of the adjacency structure of the neighborhoods. Whereas Li and Sang (2019)’s method is based on solving a fused lasso problem corresponding to a fixed spanning tree, Teixeira, Assunção, and Loschi (2019) instead developed a Gibbs sampler that sequentially samples a spanning tree, spatial partition, and regression parameters. Despite the elegance of their framework, Teixeira, Assunção, and Loschi (2019) noted that sampling a spanning tree from its exact posterior conditional distribution is computationally prohibitive. They instead sampled from an approximate conditional and the extent to which their approximation affected their downstream inference is unclear.

### 2.2 Model

**Fig. 1:**
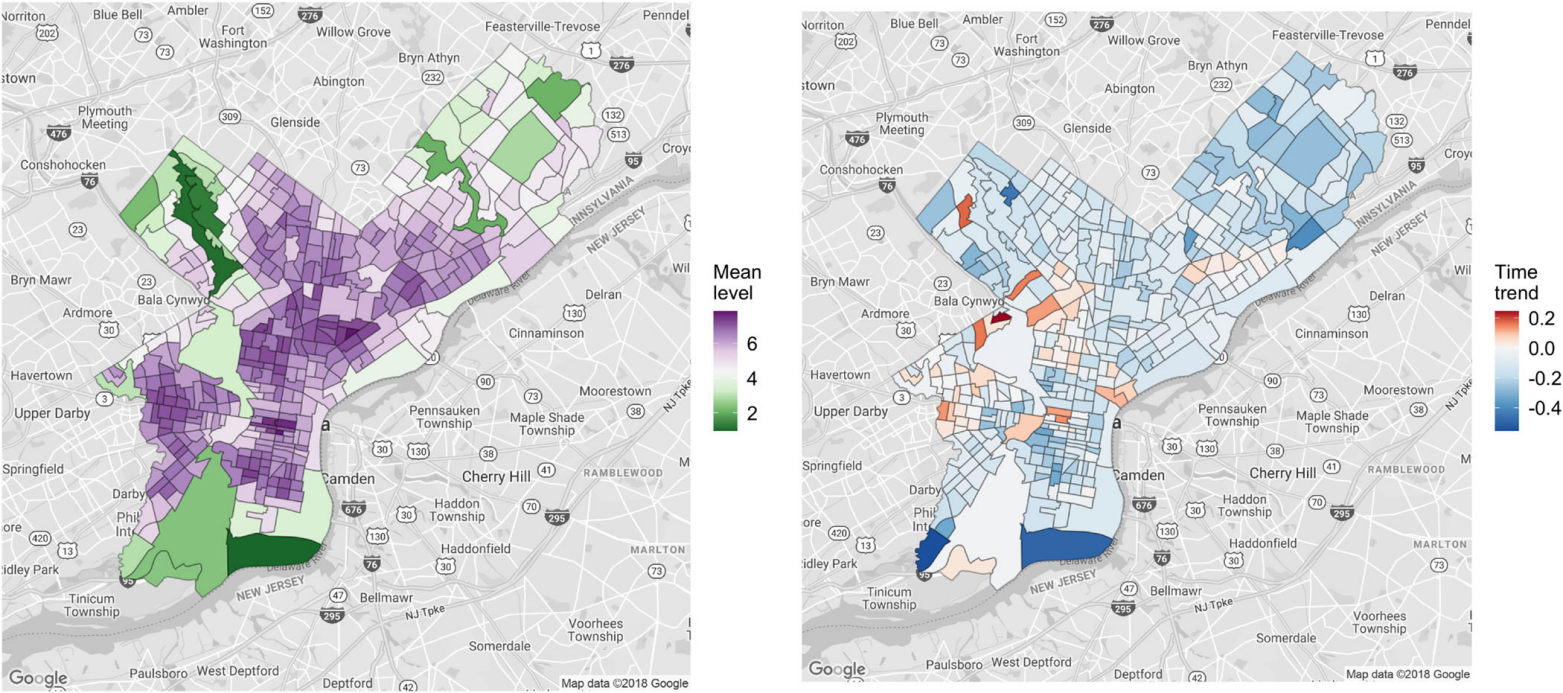
Visualization of the maximum likelihood estimates of the tract-level intercepts α (left panel) and time-trends β (right panel) for the model defined in Section 2.2.

To motivate our proposed CAR–within–cluster model, we first note that the tract-specific maximum likelihood estimates (MLEs) of *α_i_* and *β_i_* suggest that the crime may not have decreased uniformly across the city (Figure 1). In fact, in a small number of neighborhoods, crime has actually increased over the last decade.

We further observe that, with a few notable exceptions, spatially adjacent neighborhoods tend to have similar MLEs, suggesting a high degree of spatial correlation in the neighborhood-level crime dynamics. We take a hierarchical Bayesian approach in order to “borrow strength” between neighborhoods that involves specifying a prior distribution on the parameters $\alpha =(\alpha_{1},\ldots ,\alpha_{N})$ and $\beta =(\beta_{1},\ldots ,\beta_{N}).$ Because we expect the tract-specific parameters to display some spatial continuity, we use priors that explicitly introduce dependence between parameters from neighboring tracts.

Conditionally autoregressive (CAR) models are a popular class of such priors and we use a version introduced in Leroux, Lei, and Breslow (2000). Letting $W=(w_{i,j})$ be a binary adjacency matrix with $w_{i,j}=1$ if and only if neighborhoods *i* and *j* share a border, we say that the vector $\theta =(\theta_{1},\ldots ,\theta_{n})$ follows a CAR model with grand mean $\bar{\theta }$ and variance scale $\tau^{2}$ if and only if all of the full conditional distributions have the form

$$\theta_{i}|\theta_{-i},\bar{\theta },\tau^{2}∼N\left(\frac{(1-\rho )\bar{\theta }+\rho \sum_{j}w_{i,j}\theta_{j}}{1-\rho +\rho \sum_{j}w_{i,j}},\frac{\tau^{2}}{1-\rho +\rho \sum_{j}w_{i,j}}\right).$$

In this CAR model, the conditional mean of $\theta_{i}|\theta_{-i}$ is a weighted average of the grand mean $\bar{\theta }$ and the average of the *θ_j_*’s from the neighborhoods that border neighborhood *i*. The degree to which *θ_i_* is shrunk toward either of these targets is governed by a parameter ρ, which is typically set by the analyst, and the number of neighbors. These full conditionals uniquely determine the joint distribution $\theta ∼N(\bar{\theta }1_{n},\tau^{2}\Sigma_{\text{CAR}})$ where

$$\Sigma_{\text{CAR}}=\{\begin{array}{cc} {\left[\rho W^{⋆}+(1-\rho )I_{n}\right]}^{-1} & \text{if}\;n\geq 2\\ \frac{1}{1-\rho } & \text{if}\;n=1 \end{array},$$

$1_{n}$ is the *n*-vector of ones, and $W^{⋆}$ is the unweighted graph Laplacian of the adjacency matrix *W*. For compactness, we will write $\theta |\bar{\theta },\tau^{2}∼\text{CAR}(\bar{\theta },\tau^{2},W).$

Cities typically contain many geographic and social barriers like rivers and highways that manifest in sharp spatial discontinuities. In the presence of these discontinuities, a naïvely specified CAR model can induce a level of spatial smoothness among the parameters at odds with the data. To avoid this behavior, we seek *clusters* of parameters that demonstrate considerable spatial continuity within but not between clusters. We introduce two latent partitions of the neighborhoods, $\gamma^{\left(\alpha \right)}$ and $\gamma^{\left(\beta \right)}$, where $\gamma^{\left(\cdot \right)}=\{S_{1}^{\left(\cdot \right)},\ldots ,S_{K^{\left(\cdot \right)}}^{\left(\cdot \right)}\}$. We refer to the sets $S_{k}^{\left(\cdot \right)}$ as *clusters* and restrict attention to partitions consisting of clusters of spatially connected neighborhoods. We denote the set of all such partitions by SP and let $\gamma :=(\gamma^{\left(\alpha \right)},\gamma^{\left(\beta \right)})$ be the pair of latent spatial partitions underlying the mean levels and the time trends of crime across neighborhoods. In what follows, we will refer to γ as a *particle*.

To simplify our presentation, we describe only the prior over the mean levels of crime α; we place an analogous prior on the time trends β. We place independent CAR priors on the collections $\alpha_{k}=\{\alpha_{i}:i\in S_{k}^{\left(\alpha \right)}\}$, so that the joint prior density $\pi (\alpha |\gamma^{\left(\alpha \right)},\sigma^{2})$ factorizes over the collection of all clusters: $\pi (\alpha |\gamma^{\left(\alpha \right)},\sigma^{2})=\prod_{k=1}^{K^{\left(\alpha \right)}}\pi (\alpha_{k}|\sigma^{2})$. To this end, we introduce a collection of grand cluster means $\bar{\alpha }=\left\{{\bar{\alpha }}_{1},\ldots ,{\bar{\alpha }}_{K^{\left(\alpha \right)}}\right\}$ and model $\alpha_{k}|{\bar{\alpha }}_{k},\sigma^{2}∼\text{CAR}({\bar{\alpha }}_{k},a_{1}\sigma^{2},W_{k}^{\left(\alpha \right)}),$ where $W_{k}^{\left(\alpha \right)}$ is the sub-matrix of *W* whose rows and columns are indexed by the cluster $S_{k}^{\left(\alpha \right)}.$ We further place independent $N(0,a_{2}\sigma^{2})$ priors on the grand cluster means ${\bar{\alpha }}_{k}.$

We place independent truncated Ewens-Pitman priors on the two latent spatial partitions $\gamma^{\alpha }$ and $\gamma^{\beta }.$ The Ewens-Pitman distribution on partitions is equivalent to the exchangeable partition probability function (EPPF) of a Chinese Restaurant Process (Aldous 1985; Pitman 2002). It is characterized by favoring a small number of large clusters and asymptotically it induces an average of η log (N) clusters. To recover partitions with connected clusters, we truncate this distribution to the set of spatial partitions SP. The probability mass function of this prior is given by

(2) $$\pi (\gamma )\propto \eta^{K}\prod_{k=1}^{K}(n_{k}-1)!\times 1(\gamma \in SP)$$

and we denote it by T−EP. We complete our hierarchical prior with an Inverse Gamma prior on the residual variance $\sigma^{2}∼\text{IG}\left(\frac{\nu_{\sigma }}{2},\frac{\nu_{\sigma }\lambda_{\sigma }}{2}\right).$ To summarize, our model is

(3) $$\begin{array}{c} \gamma^{\left(\alpha \right)},\gamma^{\left(\beta \right)}\overset{\text{iid}}{∼}T-EP\\ \sigma^{2}∼\text{IG}\left(\frac{\nu_{\sigma }}{2},\frac{\nu_{\sigma }\lambda_{\sigma }}{2}\right)\\ {\bar{\alpha }}_{1},\ldots ,{\bar{\alpha }}_{K_{\alpha }}|\gamma^{\left(\alpha \right)},\sigma^{2}\overset{\text{iid}}{∼}N(0,a_{2}\sigma^{2})\\ {\bar{\beta }}_{1},\ldots ,{\bar{\beta }}_{K_{\beta }}|\gamma^{\left(\beta \right)},\sigma^{2}\overset{\text{iid}}{∼}N(0,b_{2}\sigma^{2})\\ \alpha_{k}|{\bar{\alpha }}_{k},\sigma^{2},\gamma^{\left(\alpha \right)}∼\text{CAR}({\bar{\alpha }}_{k},a_{1}\sigma^{2},W_{k}^{\left(\alpha \right)})\\ \;\;\text{for}\;k=1,\ldots ,K_{\alpha }\\ \beta_{k\prime }|{\bar{\beta }}_{k\prime },\sigma^{2},\gamma^{\left(\beta \right)}∼\text{CAR}({\bar{\beta }}_{k\prime },b_{1}\sigma^{2},W_{k\prime }^{\left(\beta \right)})\\ \;\;\text{for}\;k\prime =1,\ldots ,K_{\beta }\\ y_{i,t}|\alpha ,\beta ,\sigma^{2}∼N(\alpha_{i}+\beta_{i}x_{t},\sigma^{2}). \end{array}$$

The high degree of conditional conjugacy in (3) enables us to derive analytic expressions for quantities such as the marginal likelihood p(y|γ) as well as the conditional posterior expectations E[α,β|γ,y]. The availability of these expressions will be crucial for the posterior exploration strategy we develop below.

Given the residual variance $\sigma^{2}$ and latent partitions $\gamma^{\left(\alpha \right)}$ and $\gamma^{\left(\beta \right)},$ parameters in different clusters are conditionally independent. In other words, our model falls with the class of conditional product partition models (PPMs) that have been widely used in Bayesian spatial statistics (see, e.g., Knorr-Held and Raßer 2000; Denison and Holmes 2001; Feng et al. 2016). Unlike these papers, however, we are interested in recovering two latent partitions, one each for the mean levels and time-trends within each census tract. Our model is perhaps most similar to Anderson, Lee, and Dean (2017), who also seek to estimate two distinct partitions, one for the intercepts and one for the slopes. However, they limit attention to partitions containing five or fewer clusters for computational simplicity, whereas we will not need to impose any a priori restriction on the number of clusters.

Our CAR–within–cluster model depends on several hyperparameters, including *ρ*, which regulates the amount of within-cluster spatial autocorrelation, and η, which regulates the expected number of clusters. Throughout our analysis, we fix these hyperparameters and set the remaining hyperparameters $a_{1},a_{2},b_{1},b_{2},\nu_{\sigma }$, and $\lambda_{\sigma }$ in a data-dependent fashion, as described later in Section 3.2.

## 3 Posterior Exploration and Summarization

In fitting our model, we have three simultaneous aims: (i) identify the latent pair of partitions $\gamma =(\gamma^{\left(\alpha \right)},\gamma^{\left(\beta \right)}),$ (ii) estimate the approximate baseline levels α and time trends β of crime in each neighborhood, and (iii) make predictions about future incidents of crime in each neighborhood. These latter two tasks can generally be expressed as evaluating posterior expectations of the form E[g(α,β)|y] where *g* is a functional of interest. For instance, we can obtain point estimates of individual mean levels with $g(\alpha ,\beta )=\alpha_{i}$ and future crime forecasts at some future time with $g(\alpha ,\beta )=\alpha_{i}+\beta_{i}x^{⋆}.$ The combinatorial vastness of the space $SP^{2},$ which contains all possible pairs of spatial partitions, renders it impossible to enumerate all γ’s for even small values of *N*. As a result, we cannot compute the posterior probability π(γ|y) exactly.

It is tempting to resort to Markov chain Monte Carlo (MCMC) simulations to approximate expectations E[g(α,β)|y]. Unfortunately, due to the vastness of $SP^{2},$ such MCMC simulations may require a prohibitive amount of time to mix. To get around this difficulty, Anderson, Lee, and Dean (2017) arbitrarily restricted attention to partitions with no more than five clusters each. Even with such a restriction, which we will not impose, it is still quite difficult to distill the thousands of resulting draws of γ into a single point estimate and to quantify parameter and partition uncertainty in an interpretable fashion (see, e.g., Wade and Ghahramani 2018).

A popular alternative approach is posterior optimization, which usually focuses on identifying the *maximum a posteriori* (MAP) pair of partitions $\gamma^{\left(1\right)}$ or some other decision-theoretic optimal point estimate (see, e.g., Lau and Green 2007; Rastelli and Friel 2018). One then estimates the marginal expectation E[g(α,β)|y] with a “plug-in” estimator $E[g(\alpha ,\beta )|y,\gamma^{\left(1\right)}].$ Though this procedure might be substantially faster than MCMC, it completely eschews exploration of the uncertainty about γ. As a result, the MAP plug-in estimator may result in over-confident inference about the functional. Essentially, using the MAP plug-in amounts to approximating the full posterior distribution over $SP^{2}$ with a single point mass.

Instead of identifying only a single γ with high posterior probability, what if we could identify $\Gamma_{L}=\{\gamma^{\left(1\right)},\ldots ,\gamma^{\left(L\right)}\},$ the set of *L* pairs of partitions with largest posterior mass? Given $\Gamma_{L},$ a natural approximation is

$$E[g(\alpha ,\beta )|y,\gamma ]\approx \sum_{\gamma \in \Gamma_{L}}{\tilde{\pi }}_{L}(\gamma^{\left(l\right)}|y)E[g(\alpha ,\beta )|\gamma^{\left(l\right)},y],$$

where ${\tilde{\pi }}_{L}(\cdot |y)$ is the truncation of π(γ|y) to the set $\Gamma_{L}.$ Because this approximation averages over more of the posterior uncertainty about γ, we might reasonably expect it to be more accurate than the MAP plug-in.

### 3.1 A Variational Approximation

It turns out that we can identify Γ*_L_* by finding a variational approximation to the full posterior distribution. Before proceeding, we introduce some more notation. For any collection of *L* particles $\Gamma =\left\{\gamma_{1},\ldots ,\gamma_{L}\right\}$ and vector $w=(w_{1},\ldots ,w_{L})$ in the *L*-dimensional simplex, let q(·|Γ,w) be the discrete distribution that places probability $w_{l}$ on the particle $\gamma_{l}.$ Following Ročková (2018), we will refer to individual pairs of partitions γ as *particles*, the collection Γ as a *particle set* and **w** as *importance weights*. Let $Q_{L}$ be the collection of all such distributions supported on at most *L* particles. Finally, for each λ>0, let $\Pi_{\lambda }$ be the tempered marginal posterior with mass function $\pi_{\lambda }(\gamma )\propto \pi {\left(\gamma |y\right)}^{\frac{1}{\lambda }}.$ Note that the particles in $\Gamma_{L},$ which are the *L* particles with largest posterior mass, are also the *L* particles with largest tempered posterior mass for all λ.

The following proposition provides the foundation for estimating $\Gamma_{L}.$

Proposition 1 . Suppose that π(γ|y) is supported on at least *L* distinct particles and that $\pi_{\lambda }(\gamma )\neq \pi_{\lambda }(\gamma \prime )$ for γ≠γ′. Let $q_{\lambda }^{⋆}(\cdot |\Gamma^{⋆}(\lambda ),w^{⋆}(\lambda ))$ be the distribution in $Q_{L}$ that is closest to $\Pi_{\lambda }$ in a Kullback-Leibler sense:

$$q_{\lambda }^{⋆}=\text{arg}\;{\text{min}}_{q\in Q_{L}}\left\{\sum_{\gamma }q(\gamma )\;\text{log}\;\frac{q(\gamma )}{\pi_{\lambda }(\gamma )}\right\}.$$

Then $\Gamma^{⋆}(\lambda )=\Gamma_{L}$ and for each $l=1,\ldots ,L,w_{l}^{⋆}(\lambda )\propto \pi {\left(\gamma^{\left(l\right)}|y\right)}^{\frac{1}{\lambda }}$

The proof of Proposition 1 is in Section S1 of the supplementary materials and follows from the definition of the Kullback-Leibler divergence.

In light of Proposition 1, we can recover Γ*_L_* by finding an approximation of any tempered posterior $\Pi_{\lambda }.$ This is equivalent to solving

(4) $$\begin{array}{c} \left(\Gamma^{⋆}(\lambda ),w^{⋆}(\lambda )\right)\\ \;=\text{arg}\;{\text{max}}_{\left(\Gamma ,w\right)}\left\{\sum_{l=1}^{L}w_{l}\;\text{log}\;p(y,\gamma_{l})+\lambda H(\Gamma ,w)\right\}, \end{array}$$

where $H(\Gamma ,w)=-E_{q}[\text{log}\;q(\cdot |\Gamma ,w)]$ is the entropy of the approximating distribution q(·|Γ,w).

Before proceeding, we stress that we are not finding a variational approximation of $\pi (\alpha ,\beta ,\sigma^{2}|y),$ the marginal posterior distribution of the continuous parameters of interest. Instead, we are approximating the discrete posterior distribution π(γ|y), which places positive probability over all particles $\gamma =(\gamma^{\alpha },\gamma^{\beta }),$ with another discrete distribution $q^{⋆}$ that places positive probability on only *L* particles.

We pause briefly to reflect on the two terms in Equation (4). The first term is, up to an additive constant depending only on **y**, the **w**-weighted average of the height of the log-posterior at each particle in the particle set Γ. This term is clearly maximized when all of the particles in Γ are equal to the MAP. On the other hand, the entropy H(Γ,w) of the approximating distribution is maximized when all of the particles in Γ are distinct and each $w_{l}=L^{-1}.$ The penalty term *λ* balances these two opposing forces.

Finally, we note that Ročková (2018) introduced essentially the same family of optimization problems to identify sparse high-dimensional linear regression models and described a coordinate ascent strategy that iteratively updated **w** and Γ. In that work, γ was a binary vector indicating which variables to include in the model.

### 3.2 Particle Optimization

Finding the global optimum of (4) exactly is practically impossible, given the enormous size of the set of all possible particle sets Γ. Instead, like Ročková (2018), we deploy a coordinate ascent strategy: starting from an initial particle set Γ and initial weight vector w, we iteratively update one of **w** and Γ until we reach a stationary point.

To update the particle set Γ, we sweep over the individual particles, sequentially updating each one while holding the others fixed. Finding the globally optimum single particle update is practically impossible, since doing so would require searching over the space of all possible pairs of spatial partitions. We instead update each $\gamma_{l}$ by updating each of its constituent partitions one at a time. Even finding the conditional global optimum for a single partition within a single particle is practically impossible, given the vastness of the space of spatial partition SP. For that reason, we must rely on a local algorithm to search over SP. That is, we update a single partition by first enumerating a large number of candidate partitions and moving to the candidate that maximizes the objective function Equation (4).

Perhaps the simplest candidate set consists of all partitions that can be formed by reallocating a single neighborhood to a new or existing cluster. Such one-neighborhood updates directly parallel conventional Gibbs samplers for Dirichlet process mixture models. In our optimization setting, such a restrictive search strategy results in premature termination at a sub-optimal particle set Γ. Instead, a more promising strategy for navigating the space of partitions is to allow multiple neighborhoods to be reallocated at once.

**Fig. 2:**
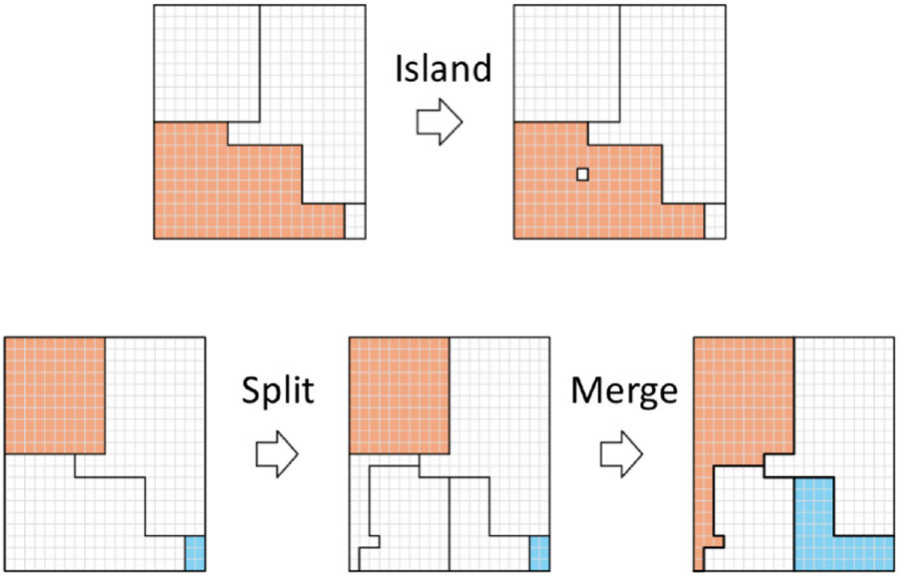
Two broad types of transitions that we consider. An “island” move (top) removes a single neighborhood from an existing cluster (the lower left orange cluster) and creates a new singleton cluster. A “split-and-merge” move (bottom) first splits an existing cluster (the bottom left cluster) into multiple sub-clusters and then merges some or all of the new sub-clusters into existing clusters.

We build a candidate set using both *fine* transitions, which reallocate a single neighborhood to a new or existing cluster, and *coarse* transitions, which simultaneously reallocate multiple neighborhoods. There are two types of fine transitions, “island” moves, in which a neighborhood is removed from its current cluster and reallocated to a new singleton cluster, and “border” moves, which move a single neighborhood located at the boundary between two clusters across the boundary. We also consider three types of coarse transitions: (i) “merge” moves, which combine two adjacent clusters into a single clusters; (ii) “split” moves, which divide an existing cluster into multiple smaller sub-clusters; and (iii) “split-and-merge” moves, which first split an existing clusters into multiple pieces and then merge some or all of those pieces with other existing clusters. Figure 2 illustrates these moves. We note that sometimes, removing a single neighborhood from a cluster leaves the resulting cluster spatially disconnected. When this happens, we treat the resulting components as individual clusters.

#### Local search heuristics

In our local search algorithm, it is not practical to enumerate all possible coarse and fine transitions. We instead use a number of heuristics to limit the number of possible moves considered in each step. For brevity, we describe these heuristics for transitions for $\gamma^{\left(\alpha \right)}$; we use exactly the same heuristics for $\gamma^{\left(\beta \right)}.$

The conditional conjugacy of our “CAR–within–cluster” model allows us to quickly compute $E[\alpha_{i}|\gamma ,y]$ and $E[{\bar{\alpha }}_{k}|\gamma ,y].$ We use these conditional means as running estimates with which to propose transitions. Rather than attempting to merge an existing cluster *k* with each one of its neighboring clusters, we only attempt to merge *k* with its neighbor k′ whose grand cluster mean ${\bar{\alpha }}_{k\prime }$ is closest to ${\bar{\alpha }}_{k}.$ Moreover, when attempting split-and-merge moves, we cap the number of new sub-clusters at five. Finally, for island moves, we initially only attempt to remove neighborhood *i* from its current cluster and move it to a new singleton if the estimated *α_i_* is in the top or bottom 5% of the distribution of estimates within the cluster. During our coordinate ascent algorithm, if we find that none of these transitions are accepted, we try all *N* island moves. This last check ensures that our algorithm converges locally in the sense that no one-neighborhood update to an individual partition will result in a higher objective.

These heuristics are admittedly ad hoc but we have found that they strike a good balance between solution optimality and computational speed. We found, for instance, that merge moves that tried to merge clusters with dissimilar estimated grand means were rarely accepted. We also found that if we restricted split moves to create only two new sub-clusters at a time, it was more difficult to identify partitions containing many small clusters.

#### Choice of hyperparameters

Our prior depends on several hyperparameters, some of which we recommend fixing and some of which we set in a data-dependent manner. Specifically, we fix ρ=0.9 so that our prior concentrates on clusters with relatively high levels of spatial autocorrelation. This choice of *ρ* also ensures prior propriety and results in somewhat more numerically stable posterior computations than values of *ρ* much closer to 1. Although the posterior distribution over γ is somewhat sensitive to *ρ*, resulting estimates of α and β are somewhat less sensitive to misspecification of *ρ* (see Section S3.4 of the supplementary materials for a detailed sensitivity analysis).

In our data analysis and simulations, the number of spatial units *N* is approximately 400. For a nontruncated Ewens-Pitman prior, the expected number of clusters grows at the rate η log (N). To us  log (400)≈6 seemed like a reasonably adequate level of summarization of crime patterns; accordingly, we fixed *η* = 1 throughout.

The remaining hyperparameters are set in a data-dependent fashion. At a high-level, to set $\nu_{\sigma }$ and $\lambda_{\sigma },$ we first fit separate linear models to the data in each neighborhood to get an empirical distribution of the residual variance. We then set the hyperparameters of the Inv. Gamma $\left(\nu_{\sigma }/2,\nu_{\sigma }\lambda_{\sigma }/2\right)$ prior on $\sigma^{2}$ to match the first and second moments of this empirical distribution. To set $a_{1},a_{2},b_{1}$, and $b_{2},$ we use an empirical Bayes approach based on computing the MAP estimate of γ. We provide full details of this strategy to Section S2 of the supplementary materials. In our experiments, we have found this strategy to work well and so have automated its implementation as a default option in our R package.

#### Initialization and choice of λ

To run our local search algorithm, one must additionally specify (i) the number of particles *L*, (ii) the initial locations of the particles, and (iii) the inverse temperature λ>0. The choice of *L* is largely dependent on the computational budget, as the per-iteration complexity of our algorithm scales linearly in *L*. For problems of our size, with around *N* = 400 spatial units, we have found that setting *L* = 10 or *L* = 20 strikes a good balance between posterior exploration and computational effort.

We initialize the particle set by randomly drawing particles $\left({\hat{\gamma }}_{K}^{\left(\alpha \right)},{\hat{\gamma }}_{K\prime }^{\left(\beta \right)}\right)$ with replacement where ${\hat{\gamma }}_{K}^{\left(\alpha \right)}$ is the partition obtained by running k-means on the maximum likelihood estimates of α with *k* = *K* clusters. We let K,K′=1,…,⌊η log (N)⌋. In this initialization, the probability of drawing particle $\left({\hat{\gamma }}_{K}^{\left(\alpha \right)},{\hat{\gamma }}_{K\prime }^{\left(\beta \right)}\right)$ is proportional to its marginal posterior probability. Our initialization allows our algorithm to pursue several search directions simultaneously but also allows for some redundancy in the initial particle set. In regions of high posterior probability, such redundancy allows multiple particles to search around a dominant mode, providing a measure of local uncertainty.

Finally, it is important to stress that *λ* is not a model parameter; neither the data likelihood nor the prior distribution depends on it. Moreover, although *λ* plays the role of a penalty parameter in Equation (4), Proposition 1 guarantees that the *global* solution to that optimization problem is the same for all λ>0. This is in marked contrast to most penalized likelihood procedures, whose solutions are highly dependent on the choice of penalty parameter. In practice, however, the solution obtained by our *local* search algorithm is somewhat sensitive to the choice of λ.

In our experiments, we have found that setting *λ* = 1 often results in all *L* particles collapsing onto a single point. On further inspection, we found that changes in the entropy, being upper bounded by  log (L), are often an order of magnitude smaller than changes in the **w**-weighted log-posterior. To offset this, we recommend running our local search algorithm with large λ. Typically, with larger values of λ, particles are encouraged to drift to regions of lower posterior probability more forcefully than with lower values of λ. In our experiments, we have found that *λ* = 10 and *λ* = 100 often produce good results.

## 4 Synthetic Data Evaluation

**Fig. 3:**
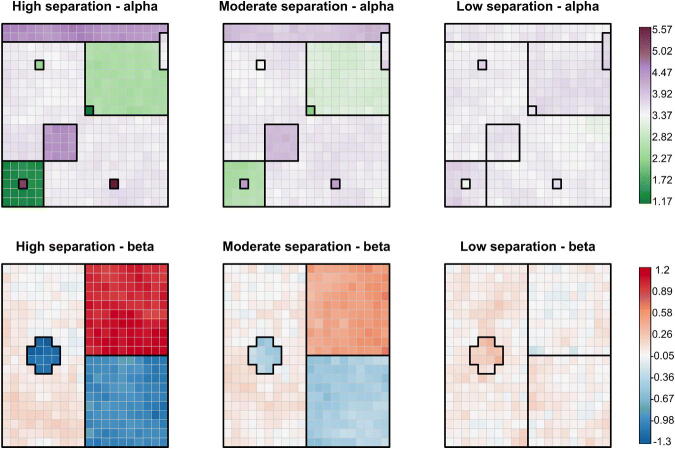
The spatial partitions used to generate our synthetic data and three different settings of α and β values. The color of each square corresponds to the value of the parameter. Different color scales have been used for α and β. Going from left to right, the distances between the cluster means get progressively smaller and the overall cluster structure is less visibly apparent.

To investigate the behavior of our proposed particle optimization procedure, which we will refer to as PartOpt throughout this section, we conducted several experiments using synthetic data generated over a 20 × 20 grid of equally-sized spatial units. Throughout our experiments, we fix the true spatial partitions ${\tilde{\gamma }}^{\alpha },$ which contains 10 clusters ranging in size from one unit to 237 units, and ${\tilde{\gamma }}^{\beta },$ which contains four clusters of sizes 188, 100, 100, and 12. Without losing any generality, we assumed throughout our simulation study that each region had unit area $A_{i}=1.$ Given the true spatial partitions, we generated synthetic data according to the model in Equation (1) where the *α_i_*’s and *β_i_*’s were drawn from CAR–within–cluster distributions. We considered three different settings, corresponding to varying levels of separation between the means ${\bar{\alpha }}_{k}$ and ${\bar{\beta }}_{k\prime }$ associated with each cluster of ${\tilde{\gamma }}^{\alpha }$ and ${\tilde{\gamma }}^{\beta }.$ This allowed us to investigate how PartOpt works in settings where the cluster structure is readily apparent and in settings where the cluster structure is less well-defined. For simplicity, although ${\tilde{\gamma }}^{\alpha }$ contains 10 distinct clusters, we fixed the corresponding cluster means ${\bar{\alpha }}_{k}$ in such a way that there were only five distinct cluster means. Figure 3 shows the true spatial partitions we used as well as a single draw of α and β in each of the three settings we considered. Full details for generating our synthetic data may be found in Section S3.1 of the supplementary materials.

We compared PartOpt method with the following competitors: (i) Anderson, Lee, and Dean (2017)’s method for finding clusters of slopes and intercepts in spatial Poisson regression (hereafter And); (ii) Li and Sang (2019)’s spatially clustered coefficient procedure (hereafter SCC); (iii) separately running K-means on the MLEs of α and β (hereafter KM); and (iv) separately running spectral clustering (Ng, Jordan, and Weiss 2002) on the MLEs of α and β (hereafter SC).

In our experiments and data analysis, we placed independent truncated Ewens-Pitman prior (2) on the latent partitions $\gamma^{\alpha }$ and $\gamma^{\beta }$ with η=1. We ran PartOpt with *L* = 10 particles and three different penalties λ∈{1,10,100}. We further fixed ρ=0.9 in the CAR–within–clusters prior and defer a sensitivity analysis of this choice of *ρ* to Section S3.4 of the supplementary materials. We moreover set the remaining hyperparameters $a_{1},a_{2},b_{1},$ and *b*_2_, together with $\nu_{\sigma }$ and $\lambda_{\sigma }$, in a data-dependent way. At a high level, we first use a heuristic based on the MLE estimate of α and β and on the expected number of clusters to estimate temporary values for the hyperparameters, with which we find the MAP; we then find an empirical Bayes estimate of the hyperparameters given the MAP and run our full procedure to recover the particle set. For further details, see Section S2 of the supplementary materials.

Note that our synthetic transformed crime densities $y_{i,t}$ are generated from the normal model in Equation (1), while And is designed for count-valued data. In order to facilitate a comparison between PartOpt and And, we first transformed our $y_{i,t}$’s into crime counts $c_{i,t}$ using the formula $c_{i,t}=b\text{sinh}(y_{i,t}+\;\text{log}\;2)e$ where bxe is the nearest integer to *x*. As noted earlier, Anderson, Lee, and Dean (2017) restricts attention to partitions with a pre-specified number of clusters. Following their recommendations we set the maximum number of clusters per partition to be five when we ran And. In Section S3.3 of the supplementary materials, we consider an additional comparison between PartOpt and And in which we first generate crime counts $c_{i,t}$ from a Poisson regression model whose slopes and intercepts are drawn from a CAR–within–clusters prior, and then compute the transformed crime densities $y_{i,t}$ using the inverse hyperbolic sine transformation.

When running KM on the MLEs of α and β, we varied the initial number of clusters from one to five and selected the final number of clusters using the silhouette method (Aranganayagi and Thangavel 2007). Since KM and And do not generally return spatially connected clusters, we post-processed the identified clusters by splitting them into their connected components. For SC, we varied the number of clusters from one to 10 and used the silhouette method to select the number of clusters. Unlike PartOpt, And, and SCC, which all attempt to learn the latent partitions jointly, KM and SC estimate $\gamma^{\alpha }$ and $\gamma^{\beta }$ essentially independently. For both KM and SC, we estimated the tract-level parameters *α_i_* and *β_i_* using the posterior mean conditional on the estimated partitions.

We compared the parameter estimation and predictive performance of these methods as well as their abilities to recover the true spatial partitions. We assessed parameter estimation using the root mean square error (RMSE) for estimating the concatenated vector of parameters ${\left(\alpha^{⊤},\beta^{⊤}\right)}^{⊤}$. We assessed predictive performance using the RMSE for predicting the vector of one-step-ahead out-of-sample observations $y_{T+1}={\left(y_{i,T+1}\right)}_{i}$ where $y_{i,T+1}∼N(\alpha_{i}+\beta_{i}x_{T+1},\sigma^{2})$. SCC, KM, and SC all estimate a single pair of partitions. We measured these methods’ ability to recover the latent spatial partitions by computing the adjusted Rand indices (Hubert and Arabie 1985) between (i) the estimated intercept partition ${\hat{\gamma }}^{\alpha }$ and the true partition ${\tilde{\gamma }}^{\alpha }$ and (ii) the estimated slope partition ${\hat{\gamma }}^{\beta }$ and the true partition ${\tilde{\gamma }}^{\beta }.$ The Rand index (Rand 1971) between two partitions is the proportion of pairs of items that are clustered together in both partitions. Values close to one indicate a high a degree of similarity between partitions. The adjusted Rand index corrects the Rand index for chance agreement.

For And, we first computed the adjusted Rand indices between each sampled partition and the corresponding true partition. We then approximated the posterior mean adjusted Rand index for each of $\gamma^{\alpha }$ and $\gamma^{\beta }.$ We similarly *approximated* the posterior mean adjusted Rand indices using the particle set identified by PartOpt. Large values of the (approximate) posterior mean adjusted Rand index indicates that the (approximate) posterior places more of its mass near the true partitions that generated the data.

**Fig. 4:**
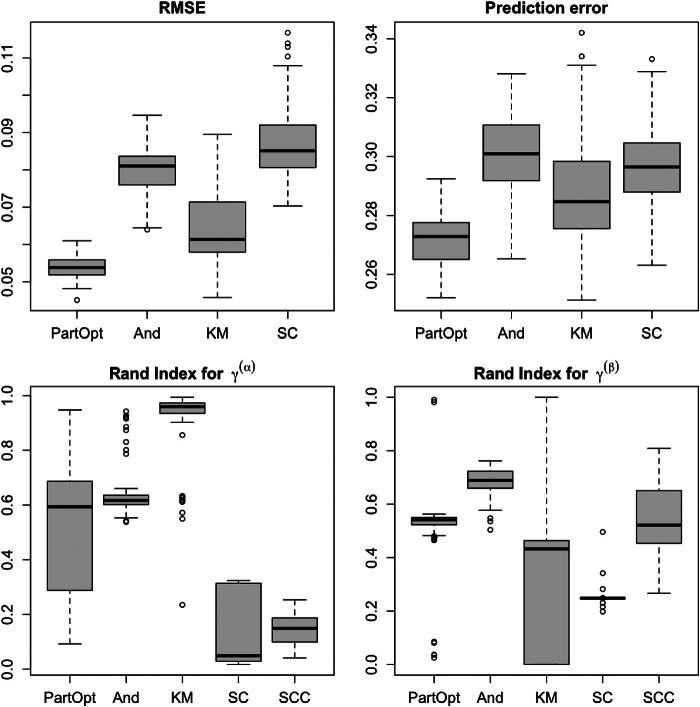
The estimation and partition selection performance of our method run with *λ* = 100 and several competitors in the moderate cluster separation setting. Across all simulations, the estimation and prediction error of SCC was substantially greater than those of other methods and are not shown.

For each of the three settings of cluster separation, we generated 100 synthetic datasets. Figure 4 shows the overall performance of the methods considered in the medium separation setting (analogous figures for the high and low separation setting may be found in Section S3.2 of the supplementary materials).

Across our simulations, PartOpt obtained consistently lower RMSE and prediction error than the competitors in each of the high, medium, and low separation settings. In all of our experiments, the estimation and prediction error of SCC was several times larger than that of every other method; indeed, in the medium separation setting, the estimation error of SCC was between 2.7 and 3.0 while the prediction error was between 4.3 and 5.4. On further inspection, we found that the clusters recovered by SCC were quite different from the clusters identified by any of the other methods. Often, SCC would cluster together spatial units with very different true parameter values, introducing substantial bias in the parameter estimates. Interestingly, while KM achieved smaller estimation and prediction error than SC in both the high and medium separation setting, SC performed better in the low separation setting, when the cluster structure was much less distinctive. As the data in our main simulation experiments was generated from a Gaussian likelihood, the Poisson regression model fit by And is mis-specified. As a result, its parameter estimates and one-step-ahead predictions are somewhat worse than those of PartOpt, which is well-specified. Interestingly, in our second set of experiments, where we generated crime counts from a Poisson regression model and then ran PartOpt on the transformed densities, PartOpt often outperformed the correctly specified And (see Section S3.3 of the supplementary materials).

In the high separation setting, each of And, KM, and PartOpt consistently recovered the true partitions. This is, in-and-of-itself, not especially surprising: in the high separation setting, the cluster structure is obvious upon visual inspection of the MLEs of α and β. Nevertheless, it is interesting to note that both SC and SCC perform quite poorly in terms of recovering the true partitions in this setting (see Figure S1 in the supplementary materials). In the medium and low separation setting, when the true cluster structure is less distinctive, the approximate posterior distribution over γ identified by PartOpt tends not to place much mass near the true partitions. On further inspection, we found that in these two settings, the true partitions that generated the data had considerably less posterior probability than the majority of the particles identified by PartOpt. This is also not especially surprising: when the data-generating parameters formed clusters which were all quite similar, the posterior favored forming a single big cluster rather than many smaller clusters with similar parameter values.

## 5 Clustering Crime Dynamics in Philadelphia

As described in Section 2, we model the transformed density of violent crimes $y_{i,t}$ in neighborhood *i* at time *t* as $y_{i,t}∼N(\alpha_{i}+\beta_{i}x_{t},\sigma^{2}).$ We further wish to identify two partitions of neighborhoods: one, $\gamma^{\left(\alpha \right)},$ that clusters together neighborhoods with similar mean levels of crime *α_i_*, and the other, $\gamma^{\left(\beta \right)},$ that clusters together neighborhoods with similar time trends $\beta_{i}.$ For our analysis of the Philadelphia crime data, we ran PartOpt using *L* = 20 particles and with λ=100. Like in our simulation experiments, we fixed the hyperparameter *η* = 1 in the truncated Ewens-Pitman priors on $\gamma^{\left(\alpha \right)}$ and $\gamma^{\left(\beta \right)}.$

### Identified particles

**Fig. 5:**
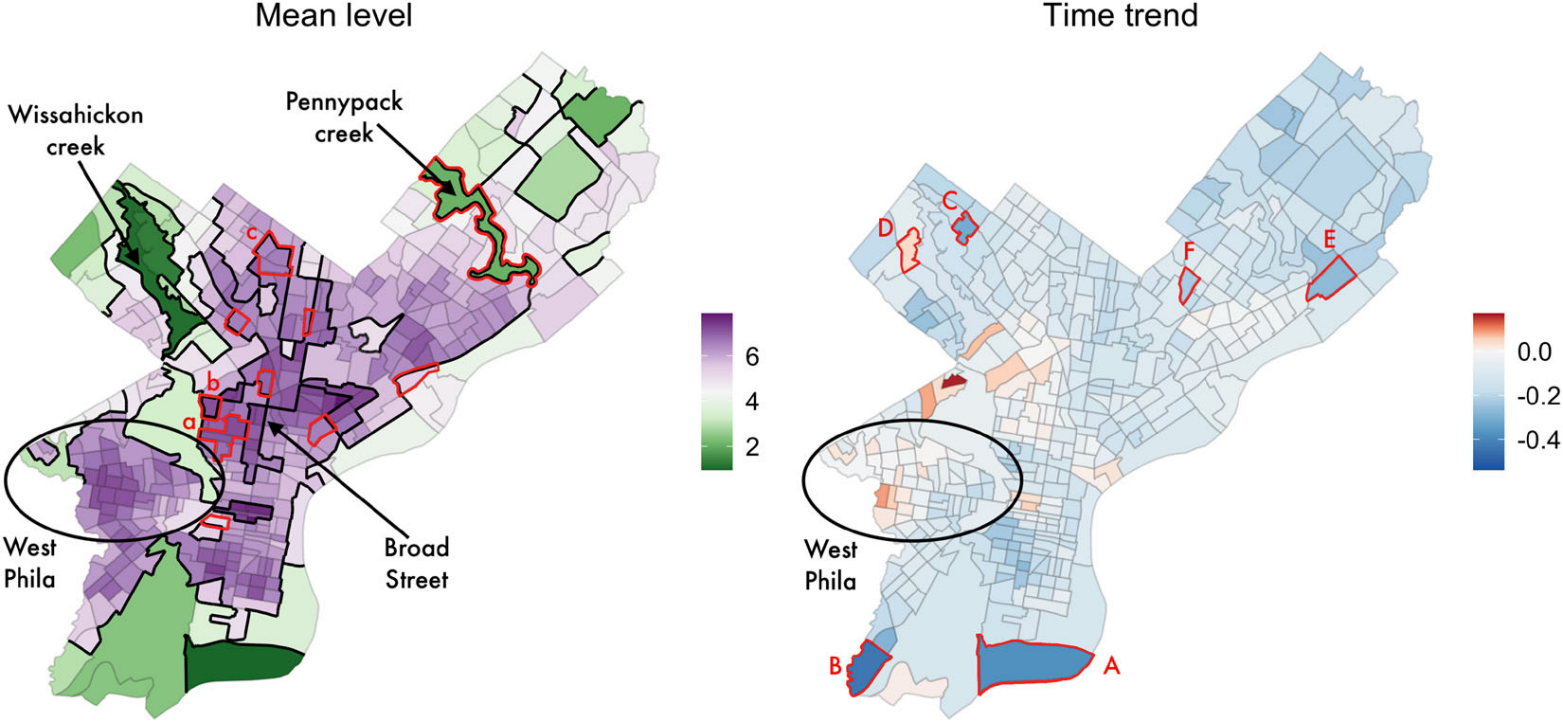
Top particle identified by PartOpt. The thick black lines delineate the borders between the clusters, and the color represents the posterior mean of α and β given the top particle. Regions highlighted in red have different cluster assignment in the particle set.

Figure 5 shows the top particle recovered by PartOpt. We highlighted the borders between clusters with thick black lines and each neighborhood’s color correspond to the conditional parameter estimate given the partition.

Immediately we notice that while the top partition for the mean level of crime density $\gamma^{\left(\alpha \right)}$ contains many clusters, the top partition for the time trends $\gamma^{\left(\beta \right)}$ contains only one cluster. We observe that it contains many neighborhoods which experienced small decreases in crime density and a handful of neighborhoods which experienced small increases in crime density. We observe that the overall range of *β_i_*’s is quite small, suggesting that the within-cluster variation is not large enough to overcome our prior’s regularization toward a small number of large clusters. Indeed, as we discuss in more detail in Section S4.3 of the supplementary materials, the posterior distribution of γ is somewhat sensitive to the choice of prior.

The remaining nineteen particles identified by PartOpt are quite similar to the top particle shown in Figure 5. In fact, the 12 particles with next highest importance weights had identical $\gamma^{\left(\beta \right)}$’s as the top particle. The particles with second through fifth highest importance weight differs from the first particle in terms of the cluster assignment of the sets of neighborhoods labeled **(a)**, **(b)**, and **(c)** in $\gamma^{\left(\alpha \right)}$, and the sixth through twelfth particles differ from the first in terms of the other neighborhoods highlighted in red.

The thirteenth through the twentieth particles have identical $\gamma^{\left(\alpha \right)}$’s, which differ from the partition shown in Figure 5 in the assignment of **(a)**, but have different $\gamma^{\left(\beta \right)}$’s. In the thirteenth particle, the neighborhood labeled **(A)** is removed from the large cluster and moved into a singleton cluster on its own. The remaining particles, respectively, separate the neighborhoods **(B)**, **(C)**, **(D)**, **(E)**, and **(F)** into their own clusters.

### Analysis of the top particle

In Figure 5, we can also immediately recognize many interesting features of Philadelphia’s built environment and geography. The clusters labeled **Wissahickon creek** and **Pennypack creek**, respectively, correspond to the parks surrounding these two rivers. In light of this, it is not especially surprising that these areas have somewhat lower baseline levels of crime than their surrounding neighborhoods. We also observe several cluster borders coinciding along with sections of the major arterial road Broad Street (labeled **Broad Street**).

Figure 5 also reveals an interesting pattern of crime dynamics in the West Philadelphia and University City region (circled in black and labeled **West Phila** in the figure). This region contains both Drexel University and the University of Pennsylvania. For the most part, the region is characterized by relatively high crime density (darker shades of purple in the left panel of the figure) with three notable exceptions on the east and southeast areas of the region. These three clusters, which are colored in lighter shades, are adjacent to university buildings and student housing. We also see a generally decreasing trend in crime in the vicinity of the universities and a slightly increasing trend further away from the universities. This finding aligns with previous reports of the positive impact of the University of Pennsylvania’s West Philadelphia Initiatives aimed at improving the social and economic landscape around the university campus (Ehlenz 2016).

### Predictive performance

Table 1 reports the root mean square error for predicting crime density in each neighborhood in 2018. We compare the performance of PartOpt, which averages over all of identified particles, with the MAP plug-in estimator. We compare it with the forecasts made by a method that does not impose any shrinkage or clustering and instead makes predictions based only on the maximum likelihood estimates of α and β (MLE); we also compare it with the competitors considered in Section 4.

**Table 1:** Out-of-sample RMSE for predicting the transformed crime density in 2018 based on models fit to data from 2006–2017.

MAP	PartOpt	MLE	And	KM	SC	SCC
0.2300	0.2299	0.2349	0.2395	0.2341	0.2401	5.970

Recall that the 20 identified particles were all quite similar to the top particle shown in Figure 5. Thus, it is not entirely surprising to see that MAP plug-in predictions are quite similar to the model averaged predictions made by PartOpt. Nevertheless, we find that by averaging over more uncertainty about the latent partitions, PartOpt achieved slightly better predictive performance. We find that And and SC perform worse than the simple MLE predictions. Interestingly, SCC recovers a single cluster of time trends *β_i_*, like the top particle shown in Figure 5. However, SCC’s estimates of the mean levels *α_i_* are substantially smaller than both the MLE and PartOpt’s estimates, yielding rather poor predictions.

## 6 Discussion

Accurate estimation of the change in crime over time is a critical first step toward a better understanding of public safety in large urban environments. An especially important challenge to such estimation is the potential presence of sharp discontinuities, which may be smoothed over by naive spatial shrinkage procedures. Focusing on the city of Philadelphia, we introduced a Bayesian hierarchical model that naturally identifies these discontinuities by partitioning the city into several clusters of neighborhoods and introduces spatial smoothness within but not between clusters. In particular, we focused on recovering two latent spatial partitions, one for the approximate baseline level of crime over the 12-year period 2006–2017 and one for the approximate time-trend.

Rather than use a computationally prohibitive stochastic search, we identified partitions with highest posterior probability by solving a single optimization problem. We showed that optimizing the proposed objective function is formally equivalent to finding a particular variational objective and introduced a local search strategy for solving this problem. While our primary focus has been on crime in the city of Philadelphia, our ensemble optimization framework is more general and there are a number of areas of future development, which we discuss below.

It is possible to run our particle optimization procedure at both higher and lower spatial resolutions. It would be interesting to run our procedure with data aggregated at the census block group level to reveal heterogeneity in crime dynamics within census tracts. Doing so would require considerably more computational resources, as there are 1336 block groups.

Though we have not done so in this article, it is also possible to adjust for important neighborhood-level covariates within our framework. For instance, one may extend Equation (1) and model $y_{i,t}∼N(\alpha_{i}+\beta_{i}x_{t}+z_{i,t}^{⊤}\theta ,\sigma^{2})$ where $z_{i,t}$ is a vector of possibly time-varying covariates. Modifying our implementation of PartOpt for this purpose is relatively straightforward with a conditionally conjugate prior on *θ*; it essentially amounts to writing a new function to compute the marginal likelihood p(y|γ). Alternatively, one could replace the Ewens-Pitman priors on the latent partitions with priors that encourage neighborhoods with similar covariates to cluster together. Many such covariate-dependent clustering priors have been introduced in the past (Park and Dunson 2010; Müller, Quintana, and Rosner 2011; Wade et al. 2014) and it would be straightforward to include support for these priors in our implementation of PartOpt. Profile regression (Molitor et al. 2010) incorporates covariates via direct regression adjustment and through the prior on clusters. It does not, however, allow different regression parameters to cluster differently nor does it permit parameters to vary within clusters. In spatial contexts, profile regression may not produce spatial clusters.

Although we have focused on identifying separate partitions of the mean levels and time trends, we believe that there are situations in which it is more desirable to identify clusters defined by both parameters. For instance, policymakers may be interested in identifying clusters of neighborhoods which have both high mean levels of crime and increasing time trends. In Section S3.5 of the supplementary materials, we discuss how we can accomplish this with a small modification to our implementation of PartOpt that constrains $\gamma^{\left(\alpha \right)}=\gamma^{\left(\beta \right)}.$

Although linear approximations to the true regression functions $f_{i,0}$ were reasonable in our dataset, in datasets with stronger suggestions of nonlinearity and more observations per census tract, it is reasonable to consider higher-order approximations of $f_{i,0}.$ With conditionally conjugate Gaussian priors on the tract-level parameters, it is still feasible to compute the marginal likelihood p(y|γ) required by particle optimization. We sketch such extensions in Section S4.2 of the supplementary materials.

Finally, while our analysis has focused on modeling crime densities, it is natural to wonder whether PartOpt can be extended to modeling crime counts with Poisson or negative binomial regressions. Our local search algorithm involves repeated calculation of the marginal likelihood p(y|γ). Unfortunately, for Poisson and negative binomial regression with CAR–within–clusters prior, these likelihoods are not available in closed form. Nevertheless, in small-scale experiments, we have found that running PartOpt with *approximate* marginal likelihoods computed using Laplace approximations works rather well. We discuss these approximations and report the preliminary results of an approximate PartOpt for Poisson regression in Section S6 of the supplementary materials.

## Supplementary Material

Supplemental Material

Supplemental Material

## Supplementary Materials

**Supplementary materials:** A manuscript containing the proof to Proposition 1, the description of the heuristic to choose hyper-parameters, additional simulation results for the synthetic evaluation of PartOpt’s performance, and an outline for extensions to the generalized linear model setting. (.pdf file)

**Github repository:** A GitHub repository containing an R package implementing our method PartOpt, code to replicate the results described in the article, analysis scripts, and instructions on how to download and aggregate the data for the analyses. (GitHub online repository).

Link: github.com/cecilia-balocchi/particle-optimization.
