# Supplementary material for: Crime in Philadelphia: Bayesian Clustering with Particle Optimization
Source: J Am Stat Assoc. 2023 Jan 18;118(542):818–29. doi: 10.1080/01621459.2022.2156348 (PMC11771475; doi:10.1080/01621459.2022.2156348)
Supplement: Supplemental Material [file UASA_A_2156348_SM8651.pdf]

JASA ACS Reproducibility Initiative - Author Contributions Checklist Form

The purpose of the Author Contributions Checklist (ACC) Form is to document the code and data supporting a manuscript, and describe how to reproduce its main results.

As of Sept. 1, 2016, the ACC Form must be included with all new submissions to JASA ACS.

This document is the initial version of the template that will be provided to authors. The JASA Associate Editors for Reproducibility will update this document with more detailed instructions and information about best practices for many of the listed requirements over time.

Data

Abstract

The data consist of the crimes reported to the Philadelphia Police, with the time, location and type of crime. We aggregate the number of violent crimes within each census tract in Philadelphia, for each year from 2006 to 2018. Additionally, the area of each census tract in Philadelphia is used to compute crime density.

Availability

Data is publicly available on opendataphilly.org. It is released by the City of Philadelphia, and the Philadelphia Police Department.

Description

Permission: The data is publicly available and its use from opendataphilly.org constitutes acceptance of the license and agreement of the City's terms of use.

- Licensing information: *The City of Philadelphia reserves all rights in the City's databases and any data contained therein, and the end user's use of the data does not constitute a transfer of, nor does the end user receive, any title or interest in the database or any other City data. The City of Philadelphia makes no representation about the accuracy of any specific information in this data and is provided "as is" and without Warranty of any kind. The user of this data will assume complete responsibility for any and all occurrences resulting from its use or display and will hold the City of Philadelphia harmless from any and all claims, demands, liabilities, obligations, damages, suits, judgments or settlements, including reasonable costs and attorneys' fees, that arise from use of this data.* (source: City of Philadelphia, Metadata catalog)
- Link to data: the data, aggregated within each year and census tract is published in Github https://anonymous.4open.science/r/particle-optimization-663E/two_partitions/data/ The sub-directory 'get_data' contains scripts and a readme file with the instructions to replicate the aggregation from the download to saving the aggregated files.
- Data provenance, including identifier or link to original data if different than above: City of Philadelphia, open data project. Links: https://www.opendataphilly.org/dataset/crime-incidents; in particular, in the CSV format, https://www.opendataphilly.org/dataset/crime-

incidents. For the 2010 Census Tracts shape files, https://www.opendataphilly.org/dataset/census-tracts

- File format: CSV and SHP
- Metadata: Available here https://www.opendataphilly.org/dataset/crime-incidents/resource/79134de9-56fa-41f2-b529-b660aaf1539b
- Version information: the data was downloaded in February 2019.

Code

Abstract

The code contains a Rcpp implementation of the particle optimization method, and the R scripts to replicate the analysis, plots and tables contained in the paper.

Description

- How delivered: the method is implemented in Rcpp.
- Licensing information: MIT license
- Link to code/repository: https://anonymous.4open.science/r/particle-optimization-663E (link and repository have been anonymized)
- Version information: Branch: master; commit: ed5643beef9e08784fefc7a6d365b3ec60440793

Optional Information

- Supporting software requirements: requires the R libraries: Rcpp, Armadillo.

Instructions for Use

Reproducibility

- What is to be reproduced: all the tables and figures from the paper, except Figure 2. How to reproduce analyses: the main code for this revised version of the paper is reported in the subdirectory "two_partitions", while the subdirectory "one_partition" contains code to replicate the simulations analysis and figures reported in Section 4 of the first submission of the manuscript.
  The main folder "two_partitions" contains the code for replication of the simulations analysis and figures reported in Section 4 of the revised manuscript, together with the analysis of the Philadelphia crime data and figures contained in Section 5.
  The simulation data can be generated using scripts/generate_simulation_data.R and the algorithm can be run using scripts/simulation.R and scripts/simulation_scc.R. These simulations were run on a high-performance computing cluster.
  For the analysis of the Philadelphia crime data, the data can be generated with get_data/create_crime_tracts2019.R and get_data/create_tracts_data.R, and the analysis with the particle optimization method was performed by running scripts/tracts_partopt.R. Additional methods were compared on this dataset using scripts/tracts_KmSc.R, scripts/tracts_anderson2017.R and scripts/tracts_scc.R.

The figures and table can be replicated with scripts/figure1.R, scripts/figure3.R, scripts/figure4.R, scripts/figure5.R and scripts/table1_predictions.R.
The subdirectory “two_partitions_laplace” contains code to reproduce the results shown in section S6 of the Supplementary Materials.

Replication

How to use software in other settings: the scripts two_partitions/scripts/tracts_partopt.R (and one_partition/scripts/illustration.R) provide examples of how the Rcpp code can be called from R.
Additionally, the subdirectory “PARTOPT” contains the source code for an R package implementing the main “PartOpt” method presented in the paper.
