# Supplementary material for: Crime in Philadelphia: Bayesian Clustering with Particle Optimization
Source: J Am Stat Assoc. 2023 Jan 18;118(542):818–29. doi: 10.1080/01621459.2022.2156348 (PMC11771475; doi:10.1080/01621459.2022.2156348)
Supplement: Supplemental Material [file UASA_A_2156348_SM8648.zip › supplement/supplement.pdf]

Supplementary Materials
Crime in Philadelphia: Bayesian Clustering with Particle Optimization

Cecilia Balocchi, Sameer K. Deshpande,

Edward I. George, and Shane T. Jensen

S1 Proof of Proposition 1

In this Section 3.1 we state that we can find the set of L particles with largest posterior by finding a variational approximation of the tempered posterior Π_λ. Here we restate Proposition 1 and provide the proof.

Remember that we denote with $\Gamma_L = \{\boldsymbol{\gamma}^{(1)}, \ldots, \boldsymbol{\gamma}^{(L)}\}$ the set of L particles with largest posterior mass, with $q(\cdot|\Gamma, \mathbf{w})$ the discrete distribution that places probability w_ℓ on the particle $\boldsymbol{\gamma}_\ell$ and with $\mathcal{Q}_L$ the collection of all such distributions supported on at most L particles. Moreover, for each $\lambda > 0$, let π_λ be the mass function of the tempered marginal posterior Π_λ, where $\pi_\lambda(\boldsymbol{\gamma}) \propto \pi(\boldsymbol{\gamma}|\mathbf{y})^{\frac{1}{\lambda}}$.

Proposition 1. *Suppose that $\pi(\boldsymbol{\gamma}|\mathbf{y})$ is supported on at least L distinct particles and that $\pi_\lambda(\boldsymbol{\gamma}) \neq \pi_\lambda(\boldsymbol{\gamma}')$ for $\boldsymbol{\gamma} \neq \boldsymbol{\gamma}'$. Let $q^\star_\lambda(\cdot|\Gamma^\star(\lambda), \mathbf{w}^\star(\lambda))$ be the distribution in $\mathcal{Q}_L$ that is closest to Π_λ in a Kullback-Leibler sense:*

$$q^\star_\lambda = \underset{q \in \mathcal{Q}_L}{\arg\min} \left\{ \sum_{\boldsymbol{\gamma}} q(\boldsymbol{\gamma}) \log \frac{q(\boldsymbol{\gamma})}{\pi_\lambda(\boldsymbol{\gamma})} \right\}.$$

Then $\Gamma^\star(\lambda) = \Gamma_L$ and for each $\ell = 1, \ldots, L$, $w^\star_\ell(\lambda) \propto \pi(\boldsymbol{\gamma}^{(\ell)}|\mathbf{y})^{\frac{1}{\lambda}}$

Proof. Denote the optimal particles $\Gamma^\star(\lambda) = \{\boldsymbol{\gamma}^\star_1, \ldots, \boldsymbol{\gamma}^\star_{L^\star}\}$. Straightforward calculus verifies

that $w^\star_\ell(\lambda) \propto \pi_\lambda(\boldsymbol{\gamma}^\star_\ell)$. We thus compute

$$\mathrm{KL}(q^\star \parallel \pi_\lambda) = \sum_{\boldsymbol{\gamma}} q^\star(\boldsymbol{\gamma}) \log \frac{q^\star(\boldsymbol{\gamma})}{\pi_\lambda(\lambda)} = -\log \Pi_\lambda(\Gamma^\star(\ell))$$

Since Π_λ is supported on at least L models, we see from this computation that if $\Gamma^\star$ contained fewer than L particles, we could achieve a lower Kullback-Leibler divergence by adding another particle $\tilde{\boldsymbol{\gamma}}$ not currently in $\Gamma^\star$ that has positive Π_λ-probability to the particle set and updating the importance weights $\mathbf{w}$ accordingly.

Now if $\Gamma^\star$ contains L models but $\Gamma^\star(\lambda) \neq \Gamma_L$, we know $\Pi_\lambda(\Gamma^\star(\lambda)) < \Pi_\lambda(\Gamma_L)$. Thus, replacing $\Gamma^\star(\lambda)$ by Γ_L and adjusting the importances weights accordingly would also result in a lower Kullback-Liebler divergence. □

S2 Various hyper-parameter choices

The main model described in Section 2 depends on several hyper-parameters, which need to be fixed by the practitioner: the parameters for the prior for σ^2 (ν_σ and λ_σ) and the multiplicative constants to specify within and between cluster variance (a_1, a_2, b_1 and b_2). We will now describe the data-dependent approach to specify such values.

Let us consider each neighborhood separately and fit a linear regression model for each one: let $\hat{\alpha}_i$ and $\hat{\beta}_i$ be the least square estimates and $\hat{\sigma}^2_i$ be the estimated residual variance for neighborhood i.

We can use the collection of $\hat{\sigma}^2_i$'s to specify the prior for σ^2: by matching mean and variance, we can recover $\nu_\sigma = 2\frac{m^2}{v} + 4$ and $\lambda_\sigma = m(1 - \frac{2}{\nu_\sigma})$, where we denote with m and v the empirical mean and variance of the $\hat{\sigma}^2_i$'s.

To specify the within and between cluster variance parameters we use a two-step heuristic: at a high level, we first find a temporary estimate of the hyperparameters a_1, a_2, b_1 and b_2, based on the MLE estimate of α_i and β_i and on the expected number of clusters; we then recover the maximum a posteriori (MAP) partition under these values and find the empirical Bayes estimate of a_1 and b_1 given the MAP. These values are finally used to run our full Particle Optimization procedure, which can be initialized from the MAP partition recovered in the first step of the heuristic.

Specifically, we consider the least square estimates $\hat{\alpha}_i, \hat{\beta}_i$, which can be thought of as an approximation of α_i, β_i given the partition with N clusters γ_N, since they do not incorporate any prior information or sharing of information; in fact under such configuration the coefficients are exchangeable and the only shrinkage induced is through the common variance parameter. Given this, one heuristic desideratum is that the marginal prior on $\boldsymbol{\alpha}|\gamma = \gamma_N$ should assign substantial probability to range of the $\hat{\alpha}_i$, assuming symmetry around zero. Specifically, we will make sure that this conditional prior places 95% of its probability over the range of the $\hat{\alpha}_i$'s. Since $\boldsymbol{\alpha}|\gamma = \gamma_N \sim N(0, \sigma^2(a_1/(1-\rho) + a_2)I_n)$, we constrain a_1 and a_2 so that

$$\frac{a_1}{1-\rho} + a_2 = \frac{\max_i |\hat{\alpha}_i|^2}{4\hat{\sigma^2}}.$$

When the MLE's are not symmetric around zero the prior probability on the range of $\hat{\alpha}_i$'s will be smaller than 95%, but at least each point in the range has prior density higher than $\phi(2)$.

In order to determine each of a_1 and a_2, we need a second constraint. To this end, consider the highly stylized setting in which we have K overlapping clusters with equal variance σ^2_{cl} whose means are equally spaced at distance $2\sigma_{cl}$. The idea of this second heuristic is to match such a stylized description to the observed distribution of $\hat{\alpha}_i$. In essence, this involves covering the range of $\hat{\alpha}_i$ with $K+1$ "chunks" of length $2\sigma_{\mathrm{cl}}$. While the exact value of σ_{cl} is unknown, we have found it useful to approximate it $a_1\sigma^2/(1-\rho)$. This approximation tends to produce smaller values of a_1, which in turn encourages a relatively larger number of clusters.

With these two constraints we can find the temporary values:

$$a_1 = \frac{(\max(\hat{\alpha}_i) - \min(\hat{\alpha}_i))^2}{4(K+1)^2\hat{\sigma}^2/(1-\rho)}$$
$$a_2 = \frac{\max_i |\hat{\alpha}_i|^2}{4\hat{\sigma^2}} - \frac{a_1}{1-\rho}.$$

Similarly for the $\hat{\beta}_i$'s we find:

$$b_1 = \frac{(\max(\hat{\beta}_i) - \min(\hat{\beta}_i))^2}{4(K+1)^2\hat{\sigma}^2/(1-\rho)}$$
$$b_2 = \frac{\max_i |\hat{\beta}_i|^2}{4\hat{\sigma^2}} - \frac{b_1}{1-\rho}.$$

In order to operationalize these heuristics, we must specify an initial guess at K. We have found in our experiments, setting $K = \lfloor \log N \rfloor$ works quite well. It, moreover, accords with the general behavior of the Ewens-Pitman prior.

We now use these values to find the MAP partition $\boldsymbol{\gamma}^{(1)}$ (we can run our Particle Optimization procedure with $L = 1$) and find the Empirical Bayes estimates of a_1 and b_1 given $\boldsymbol{\gamma}^{(1)}$ and the other hyperparameter estimates, i.e. we find

$$(\hat{a}_1, \hat{b}_1) = \arg\max_{a_1, b_1} p(Y | a_1, b_1, a_2, b_2, \nu_\sigma, \lambda_\sigma, \boldsymbol{\gamma}^{(1)})$$

using a numerical optimization algorithm.

Note that finding the MAP as part of our heuristic procedure does not increase the computational burden. In fact, even though it requires us to run the Particle Optimization algorithm twice (the first time with $L = 1$), the output from the first run can be used as a starting point in the initialization of the second run - together with the partitions recovered by running k-means on the maximum likelihood estimates. We empirically see that the MAP recovered under the temporary hyperparameters has always higher posterior probability than other initializing partitions. Consequently, when we run our Particle Optimization procedure for the second time, we start from a point with high posterior probability, harnessing the work of the initial MAP search.

S3 Additional Results: Synthetic Data Evaluation

S3.1 Synthetic data description

In Section 4, we generated several synthetic datasets based on a 20 grid of spatial units, given the true partitions $\tilde{\gamma}^{\alpha}$ and $\tilde{\gamma}^{\beta}$ and with various levels of cluster separations, as displayed in Figure 3.

We now describe in details how the synthetic data was generated. Each cluster separation setting corresponds to a pair of values $(\Delta_\alpha, \Delta_\beta)$, which are set to $(\Delta_\alpha, \Delta_\beta) = (2, 1)$ in the high separation setting, to $(\Delta_\alpha, \Delta_\beta) = (1, 0.5)$ in the moderate separation setting and to $(\Delta_\alpha, \Delta_\beta) = (0, 0)$ in the low separation setting. These values are used to construct the cluster-specific means, $\overline{\alpha}_k = 3.5 + \overline{a}_k \cdot \Delta_\alpha$ and $\overline{\beta}_k = \overline{b}_k \cdot \Delta_\beta$, where $\overline{a}_k \in \{-1, -0.5, 0, 0.5, 1\}$ and $\overline{b}_k \in \{-1, 0, 1\}$. Within each cluster, we drew the α_i's (respectively β_i's) from a CAR

model centered at a specified cluster mean with $\rho = 0.95$, hyper-parameters $a_1 = b_1 = 0.125$ and standard deviation 0.25.

Since the values of $\overline{\alpha}_k$ and $\overline{\beta}_k$ were artificially chosen and not sampled, there are no exact values for a_2 and b_2, but we can compute a lower bound for these hyperparameters so that the prior distributions of $\overline{\alpha}_k$ and $\overline{\beta}_k$ cover the values artificially chosen. If we require that the values of $\overline{\alpha}_k$ and $\overline{\beta}_k$ fall within the 0.025 and 0.975 quantiles of the prior distribution, we find that $a_2 \geq 9$ and $b_2 \geq 2$ for the high separation setting. In the moderate separation setting we find $a_2 \geq 8$ and $b_2 \geq 1$, while for the low separation setting $a_2 \geq 7$ and $b_2 \geq 0$. Note that the larger values of a_2 are due to the shift of 3.5 in the construction of $\overline{\alpha}_k$, which is important to generate data that mimics the real data which has a positive mean level of crime.

For each cluster separation setting we generated 100 pairs of vectors $(\boldsymbol{\alpha}, \boldsymbol{\beta})$. For each set of parameters we generated the outcomes $y_{i,t} \sim N(\alpha_i + \beta_i x_t, \sigma^2)$, where $x_t = (t - \mu_T)/\sigma_T$ is the time index standardized to have mean zero and unit variance. Since we used $t = 2006, \ldots, 2017$, we had $\mu_T = 2011.5$ and $\sigma_T = 3.6$.

We also considered a second data generating process, in which $c_{i,t} \sim \text{Pois}(\exp(\alpha_i + \beta_i x_t))$.

S3.2 Additional cluster separation settings

In Section 4, we compared the estimation, prediction and partition selection performance of our method to that of several competitors and we displayed results for the moderate separation setting. Specifically, for the estimation and prediction performance we displayed the root mean squared error (RMSE) for estimating the concatenated vector of parameters $(\boldsymbol{\alpha}, \boldsymbol{\beta})$ and the RMSE for the vector of one-step-ahead observations $\mathbf{y}_{T+1}$ generated from the same model. For the partition selection performance we compared the adjusted Rand index between the true partitions $\tilde{\gamma}^{\alpha}$ and $\tilde{\gamma}^{\beta}$ and the recovered one.

We now report the same measures for the additional cluster separation settings in Figure S1 and Figure S2. As in Section 4, we do not show the RMSE and Prediction error for the `SCC` method, since they were substantially greater than those of other methods (RMSE ranged between 3.9 and 4.3 in the high separation setting and between 2.4 and 3.7 in the low separation setting; prediction error ranged between 7.7 and 8.8 in the former and between 3.2 and 3.7 in the latter).

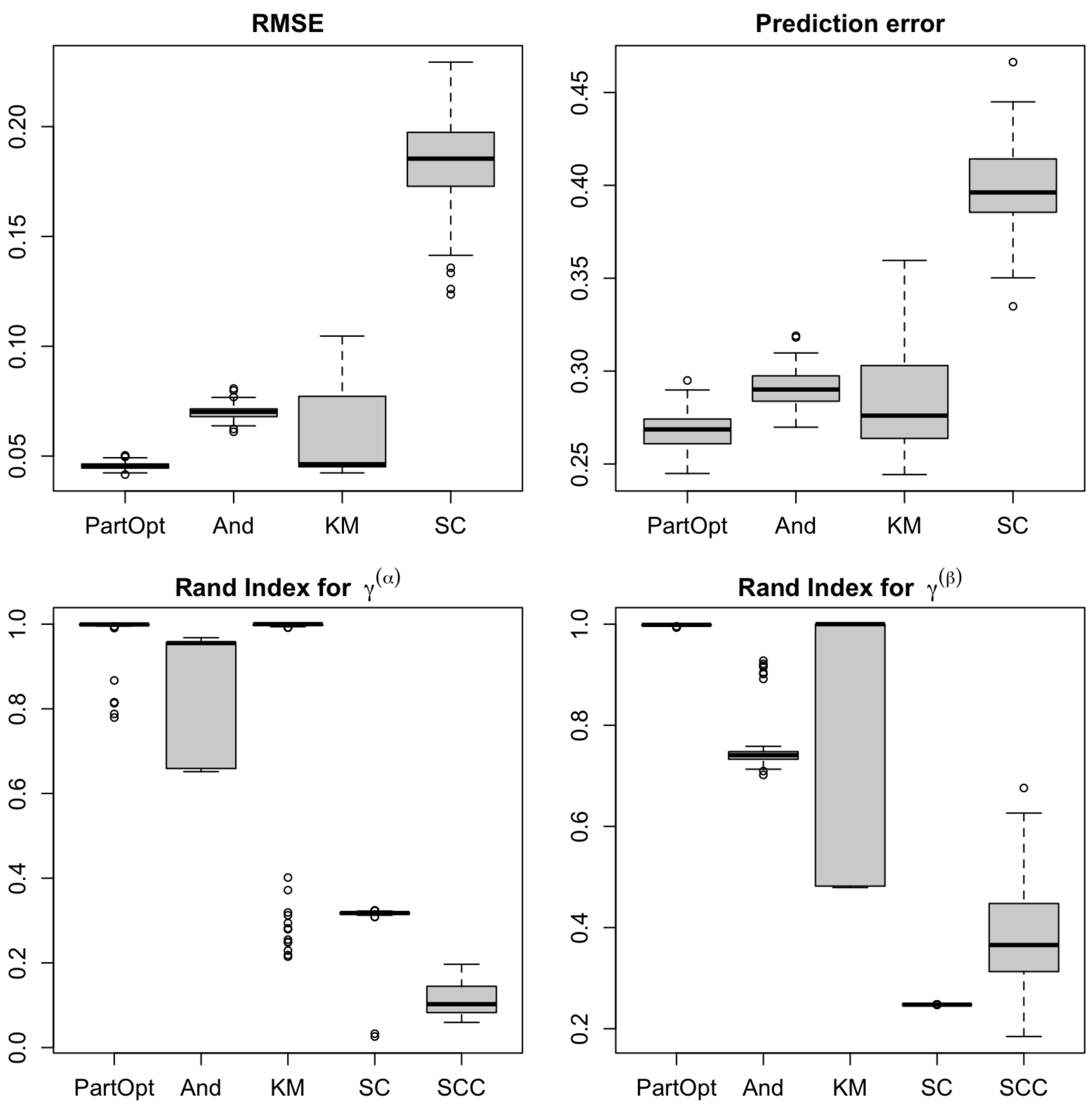

Figure S1: The estimation and partition selection performance in the high cluster separation setting.

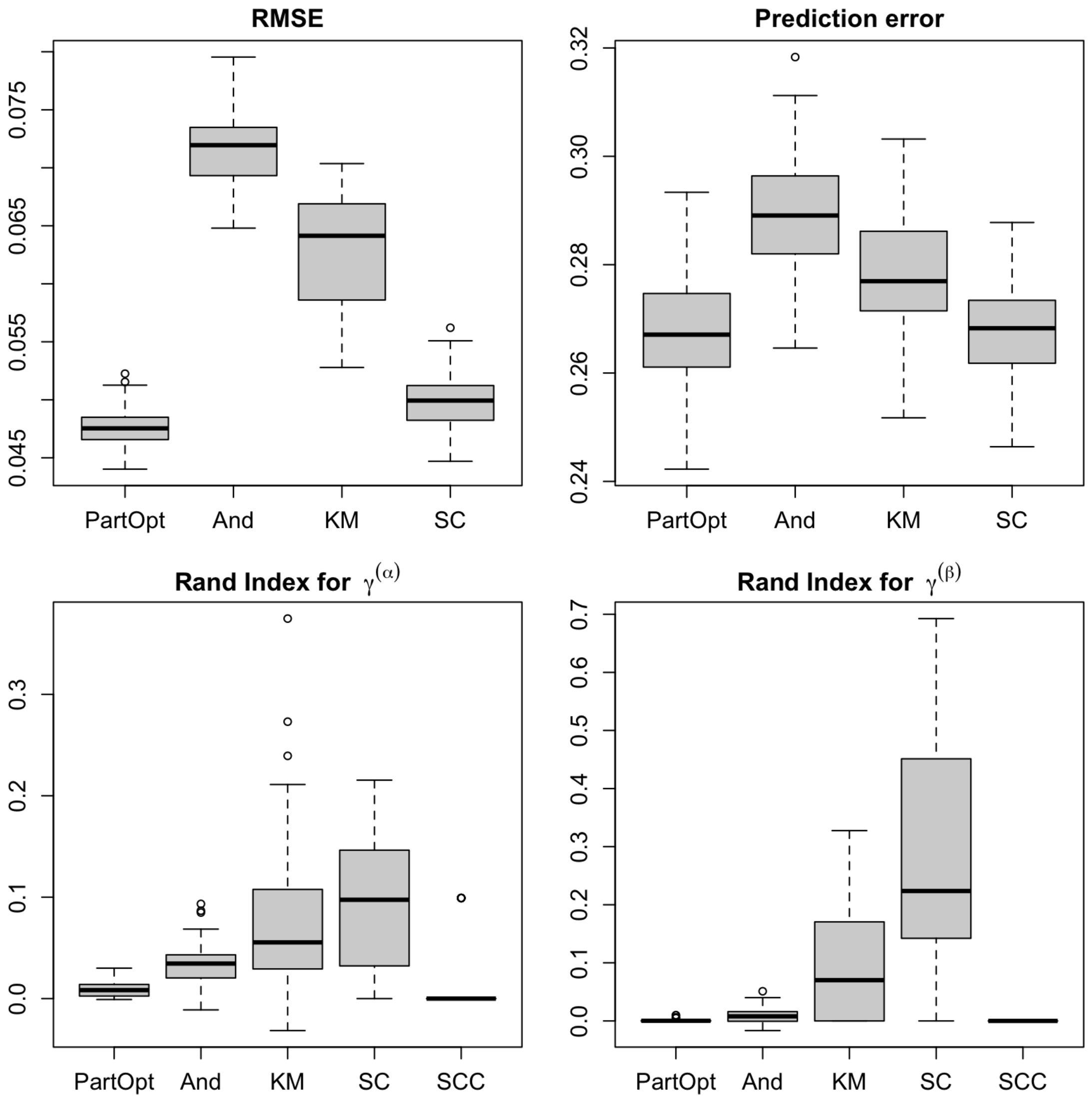

Figure S2: The estimation and partition selection performance in the low cluster separation setting.

Similarly to the moderate cluster separation setting, we see that `PartOpt` performs better in terms of estimation and predictive performance, with no other method consistently achieving second best. In fact `KM` performs almost as well as `PartOpt` in the high separation setting, while `SC` achieves second best in the low separation setting. In terms of selection performance, in the high separation setting, `PartOpt` recovers the true partitions almost always exactly, but `And` and `KM` also perform quite well; `SC` and `SCC` instead recover partitions that are quite different from the true one. In the low separation setting instead, all method have low

values for the adjusted Rand index; in fact, when there is no difference between the cluster means the true partitions $\tilde{\gamma}^\alpha$ and $\tilde{\gamma}^\beta$ lose meaning, and we expect the methods to recover the partitions with only one cluster.

To better investigate some of these issues, we report in Figures S3 some additional measures of partition selection: the log-posterior of the recovered pair of partitions, computed under the model described in Section 2, the number of clusters for the recovered γ^α (K_A) and γ^α (K_B), together with the number of clusters of the true partitions, represented as horizontal dashed lines.

It's clear from these figures that even when the adjusted Rand index is low, the log-posterior of the partitions recovered by `PartOpt` is higher than the one of the true partitions. By examining the number of clusters of the recovered partitions, we see that `SC` always underestimate the number of clusters, suggesting the reason of the poor performance we had previously noticed. `SCC` and `And` instead often overestimate the number of clusters, but for different reasons. `And` in fact does not target spatial partitions, and we have to manually find the connected components, artificially inflating the number of clusters; however, while not spatial, the partitions recovered by `And` are not so distant from the true partitions and have relatively high log-posterior values. `SCC` instead targets spatial partitions, so no manual post-processing is necessary, but it's highly sensitive to the choice of spanning tree, resulting in low values of log-posterior.

S3.3 Second data generating process

So far we have compared the behavior of `PartOpt`, `And` and the other competitors under the data generating process suggested by our model in Section 2. However, the method by Anderson et al. (2017) is developed for count data, generated from a Poisson distribution. So we also considered a second data generating process, in which the count data $c_{i,t}$ is generated from a Poisson distribution with mean $\lambda_{i,t} = \exp(\mu_{i,t})$ and $\mu_{i,t} = \alpha_i + \beta_i x_t$, and the counts are transformed, as in equation (1): $y_{i,t} = \sinh^{-1}(c_{i,t}) - \log(2)$. In Figure S4 we report the results for the simulations under this second data generating process.

In high separation settings, `And` has better estimation performance than `PartOpt` (lower RMSE and higher log-posterior), even though the latter recovers the true partitions almost exactly. However, in moderate to low separation settings, `PartOpt` performs better than `And`, both in terms of estimation performance measures (lower RMSE and prediction error) and of log-posterior of the selected partitions, suggesting that `PartOpt` is robust to misspecification

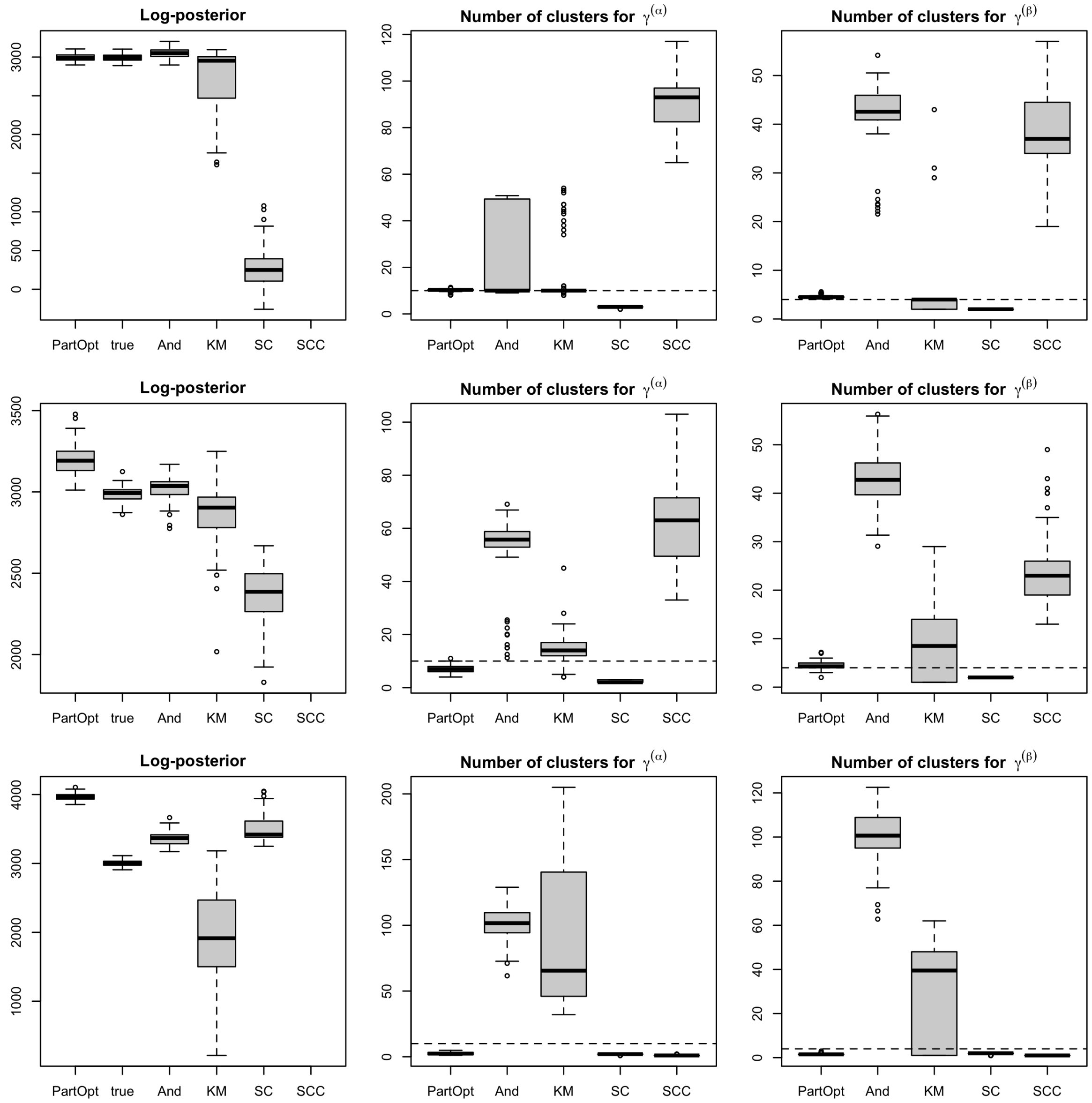

Figure S3: Additional partition selection measures in several cluster separation settings. Top row: high cluster separation. Medium row: moderate cluster separation. Bottom row: low cluster separation.

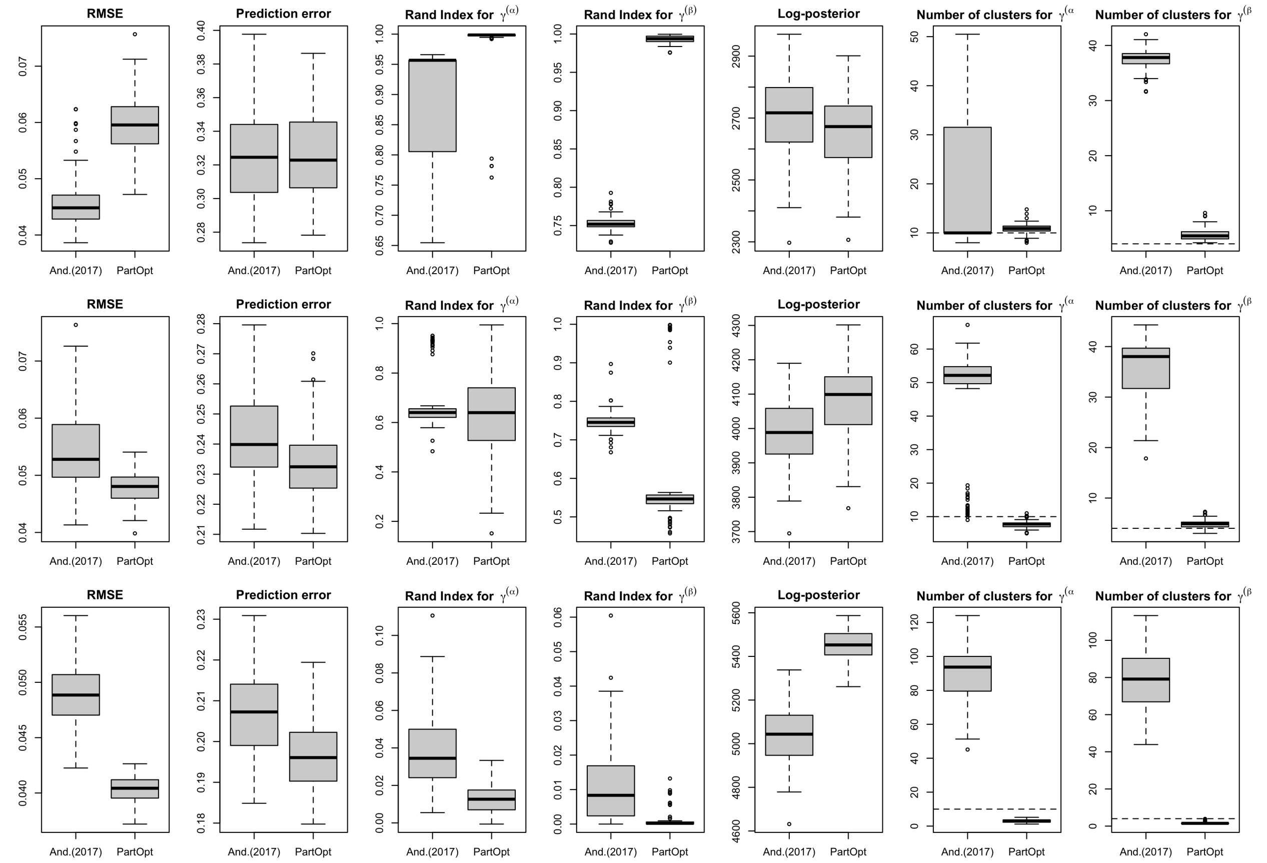

Figure S4: The estimation and partition selection performance under the second data generating process, for several cluster separation settings. Top row: high cluster separation. Medium row: moderate cluster separation. Bottom row: low cluster separation.

of the model.

S3.4 Sensitivity to hyperparameter choice

In all our simulation settings and data analysis the spatial autocorrelation hyper-parameter ρ is fixed and equal to 0.9. This choice is motivated by our search for clusters that display a large spatial autocorrelation, without having to choose an improper prior for $\boldsymbol{\alpha}$ and $\boldsymbol{\beta}$.

We now explore how the results from our synthetic analysis change when we fix a different value for the hyperparameter ρ. In particular, we consider the moderate cluster separation setting and we separately fit our model with $\rho = 0.1, 0.5, 0.75, 0.95$ together with $\rho = 0.9$ which is the value we used in our main synthetic analysis.

Figure S5 shows the estimation and partition selection performance of `PartOpt` under the various values of ρ. We first notice that there is quite some heterogeneity in the partition selection for different values of the hyperparameter ρ; in fact, for smaller values of ρ, such as $\rho = 0.1$ and $\rho = 0.5$, `PartOpt` recovers partitions that are very close to the true partitions (with high adjusted Rand index values), while for larger values of ρ it recovers partitions that are quite distant from the truth, similarly to what we discovered in our main synthetic analysis. Remember in fact that for each value of the hyperparameter the posterior distribution changes, and the particles that have largest posterior under different values of the hyperparameter will most likely not coincide. However, it is reassuring that the particle recovered by `PartOpt` under each value of the hyperparameter has almost always larger posterior probability (under such value of ρ) than the particles recovered under different values of ρ (results not shown). Moreover, for all values of the hyperparameter, `PartOpt` achieves similarly good estimation and prediction performance, suggesting robustness with respect to the choice of ρ.

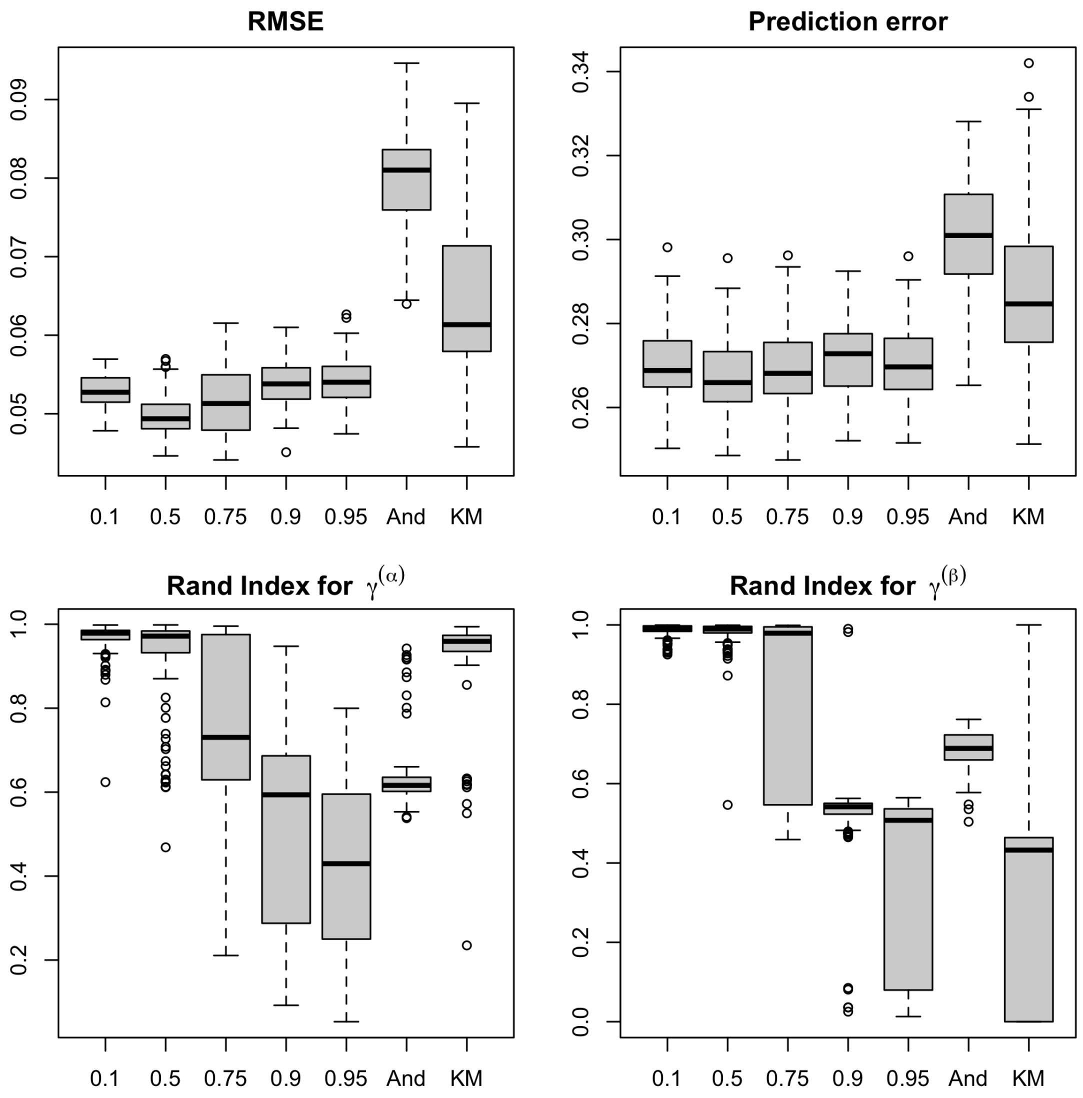

Figure S5: The estimation and partition selection performance under the moderate cluster separation setting, for several specification of the hyperparameter ρ in `PartOpt` (the labels report the value of ρ for each specification). The performance of `And` and `KM` is also reported for reference.

S3.5 Recovering equal partitions mean levels and time trends

It is possible to adapt our procedure to the case where one is interested in recovering the same partition for the mean levels and the time trends, i.e. when $\gamma^\alpha = \gamma^\beta = \gamma$. In such case, our method approximates the posterior distribution $\pi(\gamma|\mathbf{y})$ of one random partition that affects the distribution of both $\boldsymbol{\alpha}$ and $\boldsymbol{\beta}$. We have implemented this version of PartOpt

that constrains the two partitions to be equal (hereafter referred to as "Equal Partition" or `EqualPart`) and we have compared its performance to the unconstrained method that we have presented in the main manuscript, under the same synthetic data simulation described in Section S3.1. Note that for this simulation study, there two true partitions used to generate the data were different.

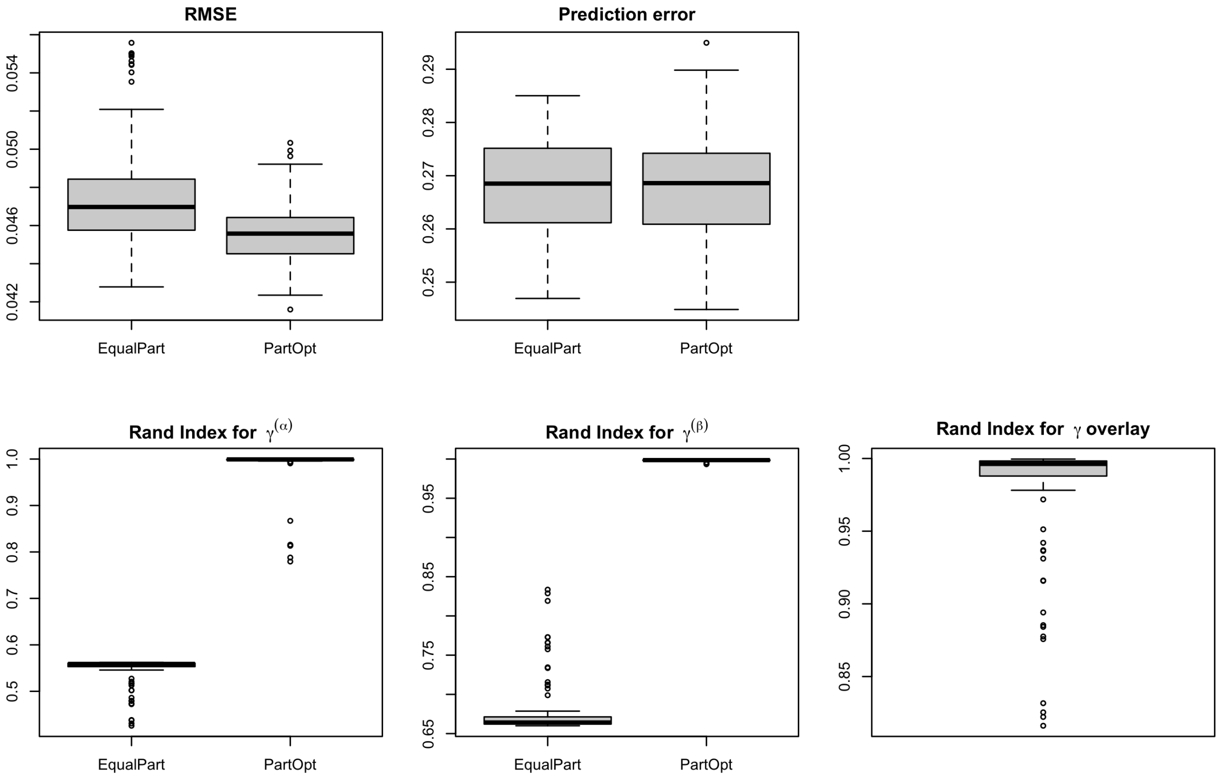

Figure S6: The estimation and partition selection performance of `equalPart` under the high cluster separation setting. The performance of `PartOpt` is also reported for reference. The panel "Rand Index for γ overlay" plots the adjusted Rand index between the partition returned by `EqualPart` and the overlay of the two true latent partitions.

Figure S6 reports the estimation and partition selection performance of `EqualPart` compared with the unconstrained `PartOpt` under the high cluster separation setting.

Interestingly, despite "Equal Partitions" being misspecified, we see that it has comparable estimation and prediction error as `PartOpt`. In terms of partition recovery, the adjusted Rand index between the partition estimated by `EqualPart` and the two partitions used to generate the data appears to be quite small. This is somewhat unsurprising: our data was

generated from a model with two latent partitions and "Equal Partitions" looks only for one. However, in virtually all of our simulation replications, the top partition recovered by `EqualPart` is quite close to the partition formed by "overlaying" the partitions in the top particle recovered by PartOpt. More specifically, this is the partition whose clusters are found as the pairwise intersection of clusters in the true partitions $\tilde{\gamma}^{\alpha}$ and $\tilde{\gamma}^{\beta}$. In the combinatorics literature this is known as the *meet* of the two partitions, which corresponds to the greatest lower bound of these partitions, under the partial order defined by the "finer than" relation. The panel "Rand Index for γ overlay" plots the adjusted Rand index between the partition returned by "Equal Partitions" and the overlay of the two true latent partitions. We see that the "Equal Partitions" routinely identified partitions close to the true overlay.

S4 Additional material on the analysis of Crime in Philadelphia

S4.1 Linearity of crime trends

In our analysis of crime in Philadelphia, we model the change of crime over time for years between 2006 and 2017 with a linear trend (see Equation (2) of the main manuscript). While linearity might not perfectly characterize the trend over time, it is the most practical and common choice when using a relatively small number of time points (see Bernardelli et al., 1995 and Anderson et al., 2017). In fact, this simple model allows us to detect the general trend, i.e. whether crime is overall increasing or decreasing in a neighborhood. However, the careful reader might worry about the validity of such assumption. We analyze here a representative sample of neighborhoods and their trend over time to check for strong non-linearities.

To analyze the linearity of crime trends we computed the Pearson correlation coefficient between time and log crime density and examined the absolute value of their correlation. Neighborhoods characterized by a correlation coefficient close to 1 (in absolute value) have trends that are very close to linear. The ones with smaller values of the correlation coefficient (again, in absolute value) are either not changing over time, or could display non-linear trend. We note that more than 50% of the neighborhoods have correlation greater than 0.6 (in absolute value) and more than 75% greater than 0.4. Figure S7 shows the trends of the 30 neighborhoods with lowest correlation in absolute value (left panel) and the 30 neighborhoods with highest correlation in absolute value (right panel). While the low correlation

neighborhoods display much more nonlinear variation than the high correlation ones, we still believe a linear trend is insightful in describing the time trend in such neighborhoods.

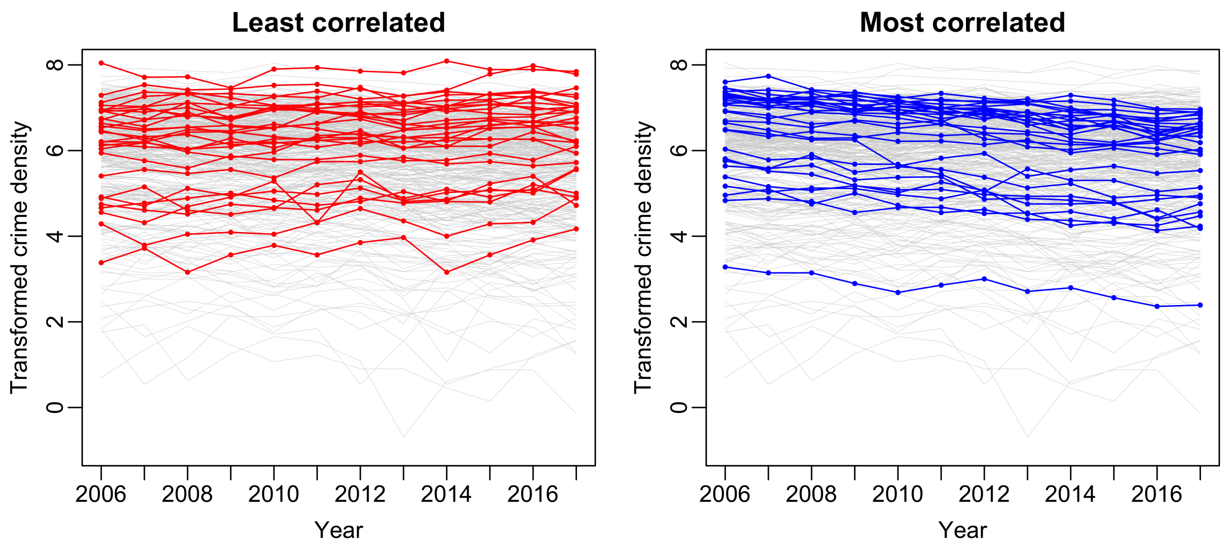

Figure S7: Trace plots for the time trends of the thirty neighborhoods with lowest absolute value of the correlation coefficient (left panel) and with the highest value of the correlation coefficient (right panel).

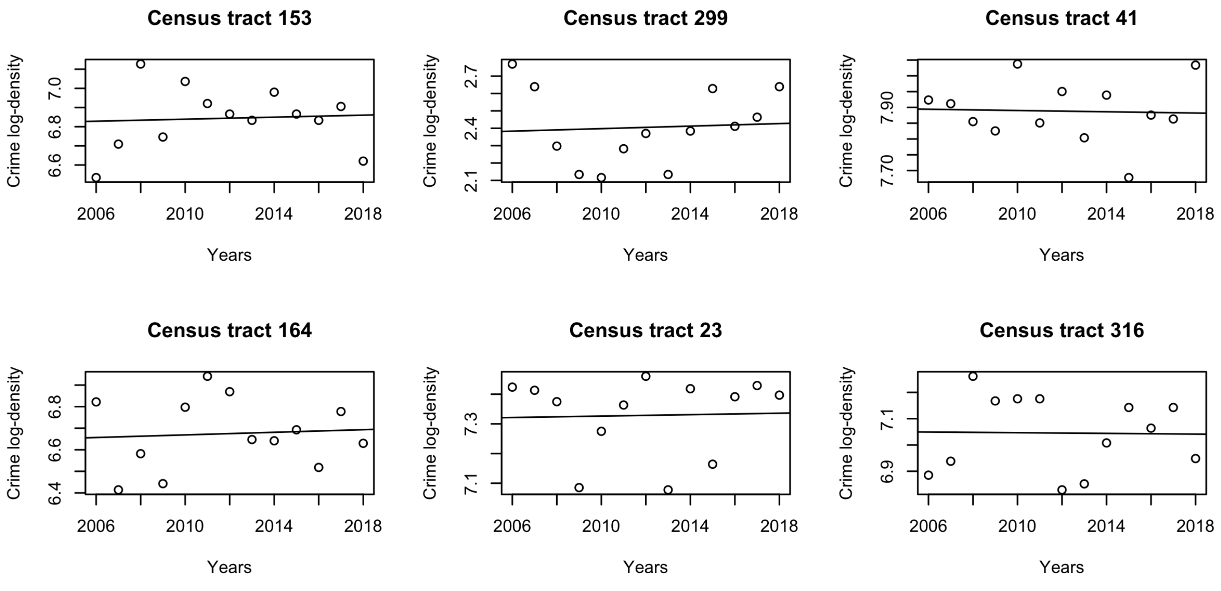

Figure S8: Crime density over time for the six neighborhoods with lowest correlation in absolute value.

To study the neighborhoods with low correlation is more detail, Figure S8 displays the time trends individually for the six neighborhoods with lowest correlation in absolute value, together with the least square line. Most of these neighborhoods do not display clear non-linear patters. The only neighborhood for which crime density seems to decrease and then increase is Census tract 299, where a quadratic term might better describe the trend.

S4.2 Extending `PartOpt` to accomodate non-linearities

In our particular application, a first-order expansion of the expected transformed crime density was sufficient to characterize the general neighborhood-level trends in crime. However, for datasets displaying strong non-linearities, such an approximate may not be appropriate. We now describe how one might extend `PartOpt` to accommodate more flexible non-linear models. To this end, consider a Dth order expansion of the model in Equation 2 of the main text:

$$y_{i,t} = \alpha_i + \sum_{d=1}^{D} \beta_{i,d} x_t^d + \varepsilon_{it}, \tag{S1}$$

where once again x_t is the standardized time index. We now must estimate the base-line transformed crime density α_i and a D-vector of regression coefficients$\boldsymbol{\beta}_i = (\beta_{i,1}, \ldots, \beta_{i,D})^\top$ for each neighborhood i.

Doing so, however, requires us to make more modeling decision than we had to make with the first-order model in the main text. Namely, we must decide how much heterogeneity we would like to allow in the spatial distributions of the coefficients in the expanded model. At one extreme, we can introduce D underlying partitions, one for each collection $\boldsymbol{\beta}^{(d)} = (\beta_{1,d}, \ldots, \beta_{N,d})$, that allows different spatial distributions for each coefficient. We could then place conditionally independent CAR–within–clusters priors on each of these collections in a manner analogous to the priors on $\boldsymbol{\alpha}$ and $\boldsymbol{\beta}$ in the main text. At the other extreme, we can introduce a single underlying partition, implicitly assuming that the spatial variability in, say, the quadratic coefficients is identical to the spatial variability in the linear coefficients. We could then place conditionally independent *multivariate* CAR (Gelfand and Vounatsou, 2003) priors on the vectors of regression coefficient for the neighborhoods in each cluster.

Although there are many possibilities for specifying the latent cluster structure of the intercepts and regression coefficients in the Dth order expansion, we may still run `PartOpt` so long as marginalize out the intercepts and regression coefficients. Essentially, so long as we adopt conditionally conjugate priors within each cluster, we can still compute the log marginal

likelihoods and conditional posterior expectations needed by `PartOpt`. We could, in fact, extend the model even further by using an alternative basis expansion in Equation (S1):

$$y_{i,t} = \alpha_i + \sum_{d=1}^{D} \beta_{i,d}\phi_d(x_t) + \varepsilon_{i,t},$$

where $\phi_1, \ldots, \phi_D$ are pre-specified basis functions. Once again, so long as we maintain conditional conjugacy within cluster, we would be able to run `PartOpt`.

S4.3 Sensitivity to prior choice

In Section 5, we analyzed the data on crime density in Philadelphia's census tracts, by running our Particle Optimization procedure on the model described in Section 2. The choice of the prior distribution and of some of the hyperparameters could affect the posterior estimates recovered. In this section, we will analyze sensitivity to prior and hyper-parameter choices. In particular, we will compare results recovered under the Ewens-Pitman prior with different η parameters and under the Uniform prior on the space of spatial partitions $\mathcal{SP}$. We additionally study the sensitivity under different values of the spatial autocorrelation parameter ρ.

We start by reporting the top particle recovered by our procedure under the Uniform prior in Figure S9. We first notice that under this prior, the partition of the time trends γ^{β} presents several clusters, identifying many moderately sized clusters that display a range of time trends, both increasing and decreasing. Moreover, the parameter estimates under the uniform prior are almost constant within cluster, in contrast to the ones under Ewens-Pitman prior that showed much larger levels of within-cluster variation. Interestingly, though we recover more clusters in the mean level partition $\gamma^{(\alpha)}$ with a uniform prior, the estimates of α_i arising from both priors show little substantive difference. While it might not be obvious from visually comparing the two plots, we can easily check by analyzing the linear correlation between the estimates of the vector of crime trends $\boldsymbol{\alpha}$ under the two priors, which is equal to 0.999. The same correlation measure on the estimates of $\boldsymbol{\beta}$ under the two models is instead 0.931, which suggests the estimates are somewhat different.

While the partition under this prior can be seen as more interpretable, it is associated with worse predictive performances: it's out of sample predictive error is 0.2344, which is larger than the error under the Ewens-Pitman prior, but smaller than the one achieved by running `And` or by finding separate MLE's (see Table 1).

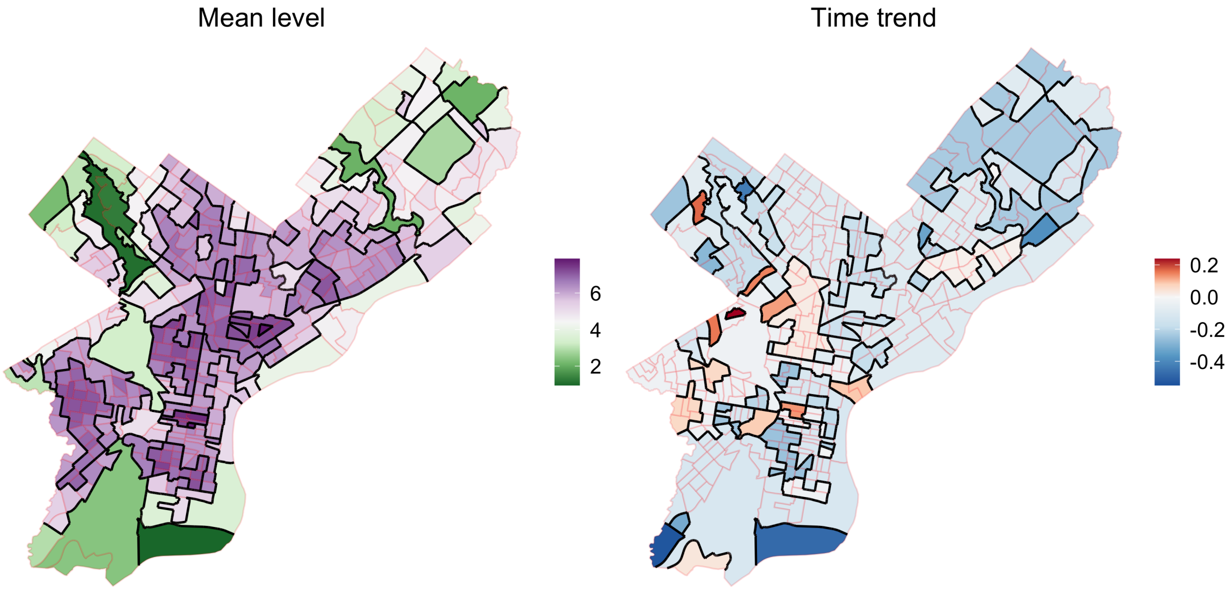

Figure S9: Top particle identified by our procedure under the Uniform prior on $\mathcal{SP}$. The thick lines highlight the border between the clusters, and the color represents the posterior mean of the parameters $\boldsymbol{\alpha}$ and $\boldsymbol{\beta}$ conditional on the displayed particle.

We now analyze the particles recovered by our procedure under the Ewens-Pitman prior with different values for the concentration hyperparameter η. This hyperparameter regulates the probability of a units joining a new cluster, with higher values inducing a larger number of clusters in expectation. Specifically we compared values $\eta = 1, 3, 5$. We find that the partition of the mean level γ^α in the top particle differs for different values of η, with the partition recovered under $\eta = 5$ displayed in Figure S10 and the one under $\eta = 3$ being very similar to it. We notice instead that the partition of the time trends γ^β does not change substantially, still showing one cluster but also recovering several singleton clusters for neighborhoods with extreme values of the time trend β_i. The change in the partition γ^α is likely caused by the local nature of our algorithm, which can get stuck in local modes. In fact, we found that the particle recovered under $\eta = 5$ has a larger posterior probability under the model with $\eta = 1$, compared to the top particle recovered under $\eta = 1$.

Finally, we considered sensitivity to the choice of the spatial autocorrelation hyperparameter ρ. In our analysis and simulation, it was chosen equal to 0.9, which induces a strong degree of spatial autocorrelation, without causing the prior distributions of $\boldsymbol{\alpha}$ and $\boldsymbol{\beta}$ to be improper (this happens when $\rho = 1$). To test the sensitivity of our procedure to this value, we ran our method with different values of ρ, ranging from low prior autocorrelations with $\rho = 0$

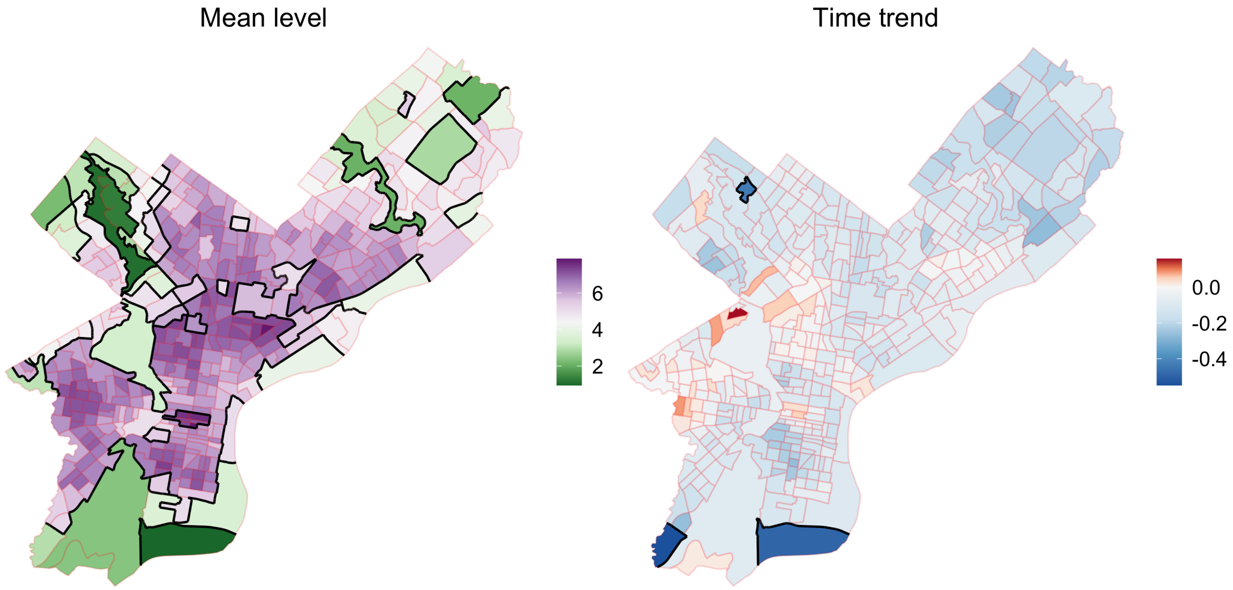

Figure S10: Top particle identified by our procedure under the Ewens-Pitman prior with concentration hyperparameter $\eta = 5$.

to very strong prior autocorrelation with $\rho = 0.99$. We analyzed the top particle recovered by our procedure. For the mean levels of crime, our procedure recovered two sets of similar partitions for different values of ρ. One set corresponds to partitions that look like the top particle displayed in Figure 5 of our main manuscript, and it was recovered for $\rho = 0.9$ and for $\rho = 0.85$. The other set of partitions (recovered for $\rho = 0.99, 0.95, 0.80, 0.75, 0.50, 0.25, 0$) is similar to the partition reported in Figure S11, which corresponds to the top particle for $\rho = 0.99$. Similarly to what we discussed previously, the difference between these two sets is likely caused by the local nature of our algorithm, which can get stuck in local modes. This seems to be the case in this example, as the partition recovered by our procedure with $\rho = 0.99$ has a higher posterior probability under the model with $\rho = 0.9$, suggesting that the algorithm with $\rho = 0.9$ and $\rho = 0.85$ got stuck in a local mode. While this is not ideal, the positive aspect is that the estimate for the $\boldsymbol{\alpha}$ values is robust to these changes, and does not show differences; in fact, the linear correlation between any pair of estimates under different ρ values is always greater than 0.99.

Instead, the partition of the time trends recovered in the top particle is always equal to the partition with one large cluster (with one exception where a singleton cluster is recovered too). In this case the estimate of the $\boldsymbol{\beta}$ values changes slightly, but not substantially: the linear correlation between the estimate found under $\rho = 0$ and the one under $\rho = 0.99$ is

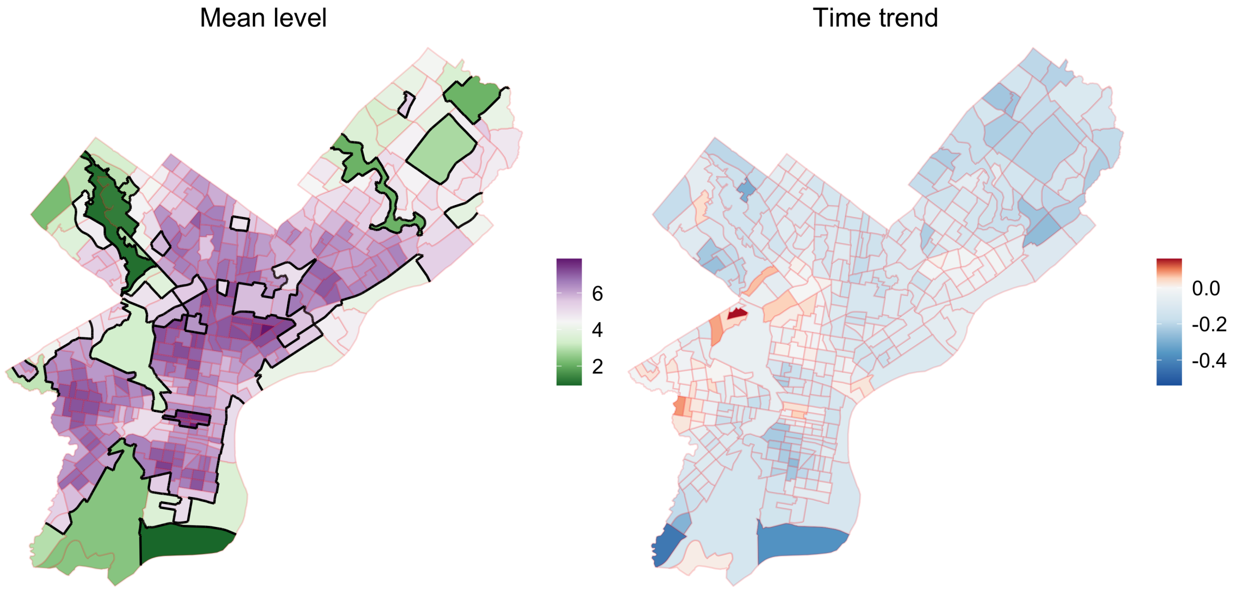

Figure S11: Top particle identified by our procedure when the spatial autocorrelation hyperparameter $\rho = 0.99$. Many of the top particles recovered in our sensitivity analysis for other values of ρ looked similar to this.

0.95 but is greater than 0.98 for any two pairs of parameters found with positive values of ρ. This effect is not surprising, as we expect the CAR prior to have stronger effects when the partition recovered is formed by only one large cluster, rather than when it is formed by many smaller clusters, as it's the case for γ^{α}.

S5 Derivation of Closed Form Expressions

Recall from Section 2 that our full model is:

$$
\begin{aligned}
\gamma^{(\alpha)}, \gamma^{(\beta)} &\sim \text{EP}(\eta; \mathcal{SP}) \\
\sigma^2 &\sim \text{IG}\left(\frac{\nu_\sigma}{2}, \frac{\nu_\sigma \lambda_\sigma}{2}\right) \\
(\overline{\alpha}_k)_k &\overset{iid}{\sim} N(0, a_2\sigma^2) \\
(\overline{\beta}_{k'})_{k'} &\overset{iid}{\sim} N(0, b_2\sigma^2) \\
(\boldsymbol{\alpha}_k)_k &\overset{ind}{\sim} \text{CAR}(\overline{\alpha}_k, a_1\sigma^2, W_k^{(\alpha)}) \\
(\boldsymbol{\beta}_{k'})_{k'} &\overset{ind}{\sim} \text{CAR}(\overline{\beta}_{k'}, b_1\sigma^2, W_{k'}^{(\beta)}) \\
(y_{i,t})_{i,t} &\overset{ind}{\sim} \text{N}(\alpha_i + \beta_i x_t, \sigma^2)
\end{aligned}
$$

We exploit the conditional conjugacy present in this model in several places. First, we have closed form expressions for the conditional posterior means $\mathbb{E}[\boldsymbol{\alpha}|\mathbf{y}, \boldsymbol{\gamma}]$ and $\mathbb{E}[\boldsymbol{\beta}|\mathbf{y}, \boldsymbol{\gamma}]$, which we use in our particle optimization procedure to propose new transitions. Second, we can compute the marginal likelihood $p(\mathbf{y}|\boldsymbol{\gamma})$ in closed form, which we use to evaluate the optimization objective and pick between multiple transitions. Below, we carefully derive these closed form expressions, noting that in several places, we can avoid potentially expensive matrix inversions. In particular, the choice to center the time variable, thereby ensuring an orthogonal design matrix within each neighborhood, facilitates rapid likelihood evaluations.

Distribution of $\boldsymbol{\alpha}_k$ Let us first consider the vector of parameters $\boldsymbol{\alpha}_k$ in cluster $S_k^{(\alpha)}$ given σ^2: by marginalizing the distribution of the grand cluster mean $\overline{\alpha}_k$, we find that its distribution is a multivariate normal with covariance matrix $\sigma^2\Sigma_k^{(\alpha)}$, where $\Sigma_k^{(\alpha)} = a_1\Sigma_{k,\text{CAR}}^{(\alpha)} + a_2\mathbf{1}\mathbf{1}^\top = a_1\left[\rho(W_k^{(\alpha)})^* + (1-\rho)\mathbf{I}\right]^{-1} + a_2\mathbf{1}\mathbf{1}^\top$. Note that its precision matrix can be computed using Woodbury's formula without having to invert any matrix:

$$
\begin{aligned}
(\Sigma_k^{(\alpha)})^{-1} &= a_1^{-1}\Omega_{k,\text{CAR}}^{(\alpha)} - a_1^{-1}\Omega_{k,\text{CAR}}^{(\alpha)}\mathbf{1}\left(a_1^{-1}\mathbf{1}^\top\Omega_{k,\text{CAR}}^{(\alpha)}\mathbf{1} + a_2^{-1}\right)^{-1}\mathbf{1}^\top a_1^{-1}\Omega_{k,\text{CAR}}^{(\alpha)} = \\
&= a_1^{-1}\Omega_{k,\text{CAR}}^{(\alpha)} - \frac{a_1^{-2}(1-\rho)^2}{a_1^{-1}n_k(1-\rho) + a_2^{-1}}\mathbf{1}\mathbf{1}^\top
\end{aligned}
$$

where $\Omega^{(\alpha)}_{k,\text{CAR}} = \left(\Sigma^{(\alpha)}_{k,\text{CAR}}\right)^{-1} = \rho(W^{(\alpha)}_k)^* + (1-\rho)\mathbf{I}$; the second line follows from noticing that $\mathbf{1}$ is both a left and right eigenvector of $\Omega^{(\alpha)}_{k,\text{CAR}}$ with eigenvalue $1-\rho$. Similarly this holds for the distribution of $\boldsymbol{\beta}_{k'}$.

Distribution of $\boldsymbol{\alpha}$ Next, we can write the distribution of the whole vector $\boldsymbol{\alpha}$ given σ^2 and $\gamma^{(\alpha)}$: by combining the distributions of the cluster specific parameters $\boldsymbol{\alpha}_k$'s, and using the independence between different clusters, we find that the distrubution of $\boldsymbol{\alpha}$ given σ^2 and $\gamma^{(\alpha)}$ is a multivariate normal with mean zero and covariance matrix that can be found by combining the $\Sigma^{(\alpha)}_k$'s. Because of the independence between clusters, *there exists an ordering of the indices of* $\boldsymbol{\alpha}$ so that the covariance matrix of $\boldsymbol{\alpha}|\gamma_\alpha, \sigma^2$ has a block-diagonal structure. We denote such permutation of the indices with $\pi^{(\alpha)}$, and it can be constructed by mapping the first n_1 elements to the indices in the first cluster ($\{\pi^{(\alpha)}(1), \dots, \pi^{(\alpha)}(n_1)\} = S^{(\alpha)}_1$), the following n_2 elements to the indices in the second cluster ($\{\pi^{(\alpha)}(n_1+1), \dots, \pi^{(\alpha)}(n_1+n_2)\} = S^{(\alpha)}_2$), and so on. With such ordering, the kth diagonal block of the covariance matrix is $\sigma^2\Sigma^{(\alpha)}_k$. Similarly, we can find a (potentially different) permutation $\pi^{(\beta)}$ for $\boldsymbol{\beta}$ and derive the distribution of $\boldsymbol{\beta}_\pi|\sigma^2, \gamma^{(\beta)}$.

Notation To describe the distributions of interest we can represent our model in the form of a unique linear model, by combining all the observations in a vector Y, combining the reodered coefficients in a unique vector $\boldsymbol{\theta} = (\boldsymbol{\alpha}_\pi, \boldsymbol{\beta}_\pi)$ and appropriately constructing the covariate matrix X. In the next paragraphs we will provide with the details on how we constructed such vectors and matrix.

To build the column vector Y we stack the vectors $\mathbf{y}_i$ with $i = 1, \dots, N$: Y is a vector of length $N \cdot T$ and each block of T rows corresponds to a particular neighborhood; in particular, the $((i-1)T + t)$th entry of Y corresponds to $y_{i,t}$.

The vector of coefficients $\boldsymbol{\theta}$ is found by concatenating the reordered $\boldsymbol{\alpha}_\pi$ and $\boldsymbol{\beta}_\pi$: for $i = 1, \dots, N$, elements $\theta_i = \alpha_{\pi^{(\alpha)}(i)}$ and $\theta_{N+i} = \beta_{\pi^{(\beta)}(i)}$.

The matrix of covariates X then has dimensions $NT \times 2N$; each block of T rows corresponds to a neighborhood and each column corresponds to an element of $\boldsymbol{\theta}$: the first N columns correspond to the elements of $\boldsymbol{\alpha}_\pi$ and the second N columns to $\boldsymbol{\beta}_\pi$. The rows of X corresponding to neighborhood i (rows $(i-1)T + t$ with $t = 1, \dots T$) have an element equal to 1 in the $(\pi^{(\alpha)})^{-1}(i)$th column, an element equal to $x_{it} = (t - \bar{t})/sd(\mathbf{t})$ in the $(N + (\pi^{(\beta)})^{-1}(i))$th column, and zero elsewhere. With such construction, the $(i-1)T + t$ row of the equation

$Y = X\boldsymbol{\theta}$ corresponds to $y_{i,t} = \theta_{(\pi^{(\alpha)})^{-1}(i)} + x_{it}\theta_{N+(\pi^{(\beta)})^{-1}(i)} = \alpha_i + x_t\beta_i$.

Marginal likelihood $Y|\gamma^{(\alpha)},\gamma^{(\beta)}$ To recover the marginal likelihood $p(Y|\gamma^{(\alpha)},\gamma^{(\beta)})$ we compute

$$\int\left[\iint p(Y|\boldsymbol{\alpha},\boldsymbol{\beta},\sigma^2)p(\boldsymbol{\alpha}|\gamma^{(\alpha)},\sigma^2)p(\boldsymbol{\beta}|\gamma^{(\beta)},\sigma^2)d\boldsymbol{\alpha}d\boldsymbol{\beta}\right]p(\sigma^2)d\sigma^2 =$$
$$=\int\left[\iint p(Y|\boldsymbol{\alpha}_\pi,\boldsymbol{\beta}_\pi,\sigma^2)p(\boldsymbol{\alpha}_\pi|\gamma^{(\alpha)},\sigma^2)p(\boldsymbol{\beta}_\pi|\gamma^{(\beta)},\sigma^2)d\boldsymbol{\alpha}_\pi d\boldsymbol{\beta}_\pi\right]p(\sigma^2)d\sigma^2 =$$
$$=\int\left[\iint p(Y|\boldsymbol{\theta},\sigma^2)p(\boldsymbol{\theta}|\gamma^{(\alpha)},\gamma^{(\beta)},\sigma^2)d\boldsymbol{\theta}\right]p(\sigma^2)d\sigma^2.$$

Let us first compute $p(Y|\sigma^2,\gamma^{(\alpha)},\gamma^{(\beta)}) = \int p(Y|\boldsymbol{\theta},\sigma^2)p(\boldsymbol{\theta}|\gamma^{(\alpha)},\gamma^{(\beta)},\sigma^2)d\boldsymbol{\theta}$. Using the notation for linear regression we can write $p(Y|\boldsymbol{\theta},\sigma^2) = N(X\boldsymbol{\theta},\sigma^2\mathbf{I})$. The prior for $\boldsymbol{\theta}$ is a normal distribution with mean zero and block covariance matrix Σ_θ: the first $n\times n$ block corresponds to the covariance matrix of $\boldsymbol{\alpha}$ and the second to the one for $\boldsymbol{\beta}$.

By integrating out $\boldsymbol{\theta}$, $p(Y|\gamma^{(\alpha)},\gamma^{(\beta)},\sigma^2) = N\left(\mathbf{0},\sigma^2\Sigma_Y\right)$ where $\Sigma_Y = \mathbf{I}+X\Sigma_\theta X^\top$. Its precision matrix can be computed using Woodbury's formula again: $\Sigma_Y^{-1} = \mathbf{I} - X(\Sigma_\theta^{-1}+X^\top X)^{-1}X^\top$. Note that $X^\top X$ is a diagonal matrix, and we derive its form towards the end of this section.

The marginal likelihood can now be derived by integrating out σ^2:

$$\begin{aligned}p(Y|\gamma^{(\alpha)},\gamma^{(\beta)}) &= \int p(Y|\sigma^2,\gamma^{(\alpha)},\gamma^{(\beta)})p(\sigma^2)d\sigma^2 = \\ &= \pi^{-nT/2}\det(\Sigma_Y)^{-1/2}\frac{(\nu_\sigma\lambda_\sigma/2)^{\nu_\sigma/2}}{\Gamma(\frac{\nu_\sigma}{2})}\int(\sigma^2)^{-\frac{NT+\nu_\sigma}{2}-1}e^{-\frac{Y^\top\Sigma_Y^{-1}Y+\nu_\sigma\lambda_\sigma}{2\sigma^2}}d\sigma^2 = \\ &= \pi^{-nT/2}\det(\Sigma_Y)^{-1/2}\frac{\Gamma(\frac{NT+\nu_\sigma}{2})}{\Gamma(\frac{\nu_\sigma}{2})}\left(\frac{\nu_\sigma\lambda_\sigma}{2}\right)^{\nu_\sigma/2}\left(\frac{\nu_\sigma\lambda_\sigma+Y^\top\Sigma_Y^{-1}Y}{2}\right)^{-(NT+\nu_\sigma)/2} = \\ &= \pi^{-nT/2}\det(\Sigma_Y)^{-1/2}\frac{\Gamma(\frac{NT+\nu_\sigma}{2})}{\Gamma(\frac{\nu_\sigma}{2})}\left(\frac{\nu_\sigma\lambda_\sigma}{2}\right)^{-NT/2}\left(1+\frac{Y^\top\Sigma_Y^{-1}Y}{\nu_\sigma\lambda_\sigma}\right)^{-(NT+\nu_\sigma)/2}.\end{aligned}$$

Note that if $\lambda_\sigma = 1$, this is multivariate t-distribution with ν_σ degrees of freedom.

For this we need to compute the quadratic form

$$Y^\top\Sigma_Y^{-1}Y = Y^\top Y - Y^\top X(\Sigma_\theta^{-1}+X^\top X)^{-1}X^\top Y.$$

Because of the block diagonal structure of $\Sigma_\theta^{-1}+X^\top X$ we can write this as a sum over the

clusters of the two partitions. Consider the column vector $X^\top Y$ of length $2N$: the first N elements correspond to the summary statistics related to the $\alpha_{\pi(i)}$'s and we will denote the ones corresponding to cluster $S_k^{(\alpha)}$ with $(X^\top Y)_k^{(\alpha)}$, while the second N elements are for the β_i's and we denote with $(X^\top Y)_{k'}^{(\beta)}$ the ones for cluster $S_{k'}^{(\beta)}$. Now we can write

$$\begin{aligned}Y^\top X(\Sigma_\theta^{-1} + X^\top X)^{-1} X^\top Y = &\sum_{k=1}^{K^{(\alpha)}} (X^\top Y)_k^{(\alpha)\top} ((\Sigma_k^{(\alpha)})^{-1} + T\mathbf{I})^{-1} (X^\top Y)_k^{(\alpha)} \\ &+ \sum_{k'=1}^{K^{(\beta)}} (X^\top Y)_{k'}^{(\beta)\top} ((\Sigma_{k'}^{(\beta)})^{-1} + \sum x_t^2 \mathbf{I})^{-1} (X^\top Y)_{k'}^{(\beta)}\end{aligned}$$

where $(\Sigma_k^{(\alpha)})^{-1} + T\mathbf{I}$ is the diagonal blocks of $\Sigma_\theta^{-1} + X^\top X$ corresponding to cluster $S_k^{(\alpha)}$ and $(\Sigma_{k'}^{(\beta)})^{-1} + \sum x_t^2 \mathbf{I}$ corresponds to $S_{k'}^{(\beta)}$; each of them can be inverted using methods for symmetric positive definite matrices.

To compute the marginal likelihood we are left we calculating the determinant of Σ_Y, where we can use the reciprocal of the determinant of its inverse

$$\det(\Sigma_Y^{-1}) = \det(\mathbf{I} - X(\Sigma_\theta^{-1} + X^\top X)^{-1} X^\top) = \det(\mathbf{I} - (\Sigma_\theta^{-1} + X^\top X)^{-1} X^\top X)$$

where the last equality is given by Sylvester's formula, and allows us to compute the determinant of a smaller dimensional matrix. Moreover, because of its block diagonal structure, we can compute the determinant block-wise.

Posterior mean of $\boldsymbol{\alpha}, \boldsymbol{\beta}$ The calculations for the posterior mean of $\boldsymbol{\alpha}, \boldsymbol{\beta}$ are very similar: using the same notation and the results for linear regression, we can find

$$\mathbb{E}\left[\boldsymbol{\theta} | Y, \gamma^{(\alpha)}, \gamma^{(\beta)}, \sigma^{-1}\right] = \left(X^\top X + \Sigma_\theta^{-1}\right)^{-1} X^\top Y$$

and since this does not depend on σ^2, it coincides with $\mathbb{E}\left[\boldsymbol{\theta} | Y, \gamma^{(\alpha)}, \gamma^{(\beta)}\right]$. Because of the block diagonal structure of the matrices involved, we can compute the estimate of the parameter for each cluster independently. Moreover, note that the inverse of $X^\top X + \Sigma_\theta^{-1}$ is computed in the likelihood calculation, so it can be stored and does not need to be computed two times.

Derivation of $X^\top X$ Since in our formulation the covariates are orthogonal, i.e. $\sum_{t=1}^T x_{it} = 0$ for all i, $X^\top X$ is a diagonal matrix. Note that column $X_{(\pi^{(\alpha)})^{-1}(i')}$ contains T 1's in rows

$t + (i' - 1)\times$T and zeros elsewhere; similarly column $X_{N+(\pi^{(\beta)})^{-1}(i')}$ contains elements $(x_{i't})$ in rows $t + (i' - 1) \times T$ and zero's elsewhere. Thus, when we compute $(X^\top X)_{ij}$ we consider the cross product of columns X_i and X_j. Depending on the value of i and j, we have the following cases:

- if $i = j \leq N$, then $(X^\top X)_{ij} = T$,
- if $i = j \geq N$, then $(X^\top X)_{ij} = \sum_t x^2_{\pi^{(\beta)}(j-N),t}$,
- if $i \leq N$ and $j = N + i$, then $(X^\top X)_{ij} = \sum_t x_{\pi^{(\beta)}(i),t} = 0$,
- if $j \leq N$ and $i = N + j$, then $(X^\top X)_{ij} = \sum_t x_{\pi^{(\beta)}(j),t} = 0$,
- for any other i, j, $(X^\top X)_{ij} = 0$.

Thus the matrix $X^\top X$ is a diagonal matrix: the first $n \times n$ diagonal block is $T\mathbf{I}$, and the second diagonal block is a diagonal matrix whose entries are $\sum_{t=1}^T x^2_{it}$; when we have fixed design, $x_{it} = x_t = (t - \bar{t})/sd(\mathbf{t})$, then $\sum_{t=1}^T x^2_{it} = \sum_{t=1}^T((t - \bar{t})/sd(\mathbf{t}))^2$ is constant, so the second diagonal block is $\sum x^2_{it}\mathbf{I}$. Because of the orthogonality of the covariates, the upper-right and lower-left blocks are zero matrices, since $\sum_{t=1}^T x_{it} = 0$.

Note on cluster-wise update of calculations. In our greedy search when we perform a move only one or two clusters in only one partition is changed: in a *split* move for $\gamma^{(\cdot)}$, a cluster is divided into two sub-clusters, and the original cluster replaced by the first, while the second creates an additional cluster; in a *merge* move, one of two clusters is deleted and the other is replaced to the merge of the two original clusters. In each case, we need to update the value of the marginal likelihood, of the prior for $\gamma^{(\cdot)}$ and of the estimate of the parameters.

Because of the block structure given by orthogonality of covariates and by the reordering of the parameters, changing the structure of some clusters does not affect the parameter estimates for other clusters that are not involved in the move. This implies that updates for updates to $S_k^{(\alpha)}$ do not affect the parameter estimates $\boldsymbol{\alpha}_h$ for $h \neq k$ or $\boldsymbol{\beta}_{k'}$ for any k'. Similarly, since the quadratic form $Y^\top\Sigma_Y^{-1}Y$ can be written as sum of cluster-specific quadratic forms, we can update only the quadratic form of the clusters affected and we can compute the determinant of the blocks of Σ_Y corresponding to the modified clusters.

This allows us to invert matrices that scale like the size of the clusters, reducing the computational costs dramatically.

S6 Extension to Non-Conjugate Models

The proposed particle optimization strategy relies on the ability to compute the marginal likelihood $\pi(\mathbf{y}|\boldsymbol{\gamma})$. This is often straightforward with conjugate models, such as the one considered for our application. However, it may be challenging or impossible to compute $\pi(\mathbf{y}|\boldsymbol{\gamma})$ in more complicated non-conjugate settings. We can, nevertheless, *approximate* the marginal likelihood using, e.g., a Laplace approximation and deploy an approximate particle optimization strategy. Below, we outline how this works for Poisson regression.

For example, consider a Poisson regression model for count data, $c_{it} \sim \text{Pois}\left(\exp\left\{\alpha_i + \beta_i x_t\right\}\right)$, with separate CAR-within-clusters priors on the α_i's and β_i's. That is, similar to model (3) in the main text, we model

$$
\begin{aligned}
\gamma^{(\alpha)}, \gamma^{(\beta)} &\overset{iid}{\sim} \mathcal{T}\text{-}\mathcal{EP} \\
\sigma^2 &\sim \text{IG}\left(\frac{\nu_\sigma}{2}, \frac{\nu_\sigma \lambda_\sigma}{2}\right) \\
\overline{\alpha}_1, \ldots, \overline{\alpha}_{K_\alpha} | \gamma^{(\alpha)}, \sigma^2 &\overset{iid}{\sim} N(0, a_2\sigma^2) \\
\overline{\beta}_1, \ldots, \overline{\beta}_{K_\beta} | \gamma^{(\beta)}, \sigma^2 &\overset{iid}{\sim} N(0, b_2\sigma^2) \\
\boldsymbol{\alpha}_k | \overline{\alpha}_k, \sigma^2, \gamma^{(\alpha)} &\sim \text{CAR}(\overline{\alpha}_k, a_1\sigma^2, W_k^{(\alpha)}) \quad \text{for } k = 1, \ldots, K_\alpha \\
\boldsymbol{\beta}_{k'} | \overline{\beta}_{k'}, \sigma^2, \gamma^{(\beta)} &\sim \text{CAR}(\overline{\beta}_{k'}, b_1\sigma^2, W_{k'}^{(\beta)}) \quad \text{for } k' = 1, \ldots, K_\beta \\
c_{i,t} | \boldsymbol{\alpha}, \boldsymbol{\beta} &\sim \text{Pois}(\exp(\alpha_i + \beta_i x_t))
\end{aligned}
\tag{S2}
$$

where x_t corresponds to the time index standardized to have mean zero and unit variance.

In the main text, we defined a particle to be the pair of partitions $(\gamma^{(\alpha)}, \gamma^{(\beta)})$. Now, we extend the definition of particle to include σ^2; that is, let $\boldsymbol{\gamma} = (\gamma^{(\alpha)}, \gamma^{(\beta)}, \sigma^2)$. To approximate the posterior distribution $\pi(\boldsymbol{\gamma}|\mathbf{y})$, we first approximate the marginal likelihood $\pi(\mathbf{y}|\boldsymbol{\gamma})$. Using a Laplace approximation, we can compute

$$
\pi(\boldsymbol{\gamma}|\mathbf{y}) = \frac{\pi(\boldsymbol{\gamma}, \boldsymbol{\alpha}, \boldsymbol{\beta}|\mathbf{y})}{\pi(\boldsymbol{\alpha}, \boldsymbol{\beta}|\mathbf{y}, \boldsymbol{\gamma})} \simeq \left.\frac{\pi(\boldsymbol{\gamma}, \boldsymbol{\alpha}, \boldsymbol{\beta}|\mathbf{y})}{\hat{\pi}(\boldsymbol{\alpha}, \boldsymbol{\beta}|\mathbf{y}, \boldsymbol{\gamma})}\right|_{(\boldsymbol{\alpha},\boldsymbol{\beta}) = (\hat{\boldsymbol{\alpha}}, \hat{\boldsymbol{\beta}})}
\tag{S3}
$$

where $\hat{\pi}(\boldsymbol{\alpha}, \boldsymbol{\beta}|\mathbf{y}, \boldsymbol{\gamma})$ is the density of a multivariate normal distribution whose mean is equal to the conditional MAP estimate $(\hat{\boldsymbol{\alpha}}, \hat{\boldsymbol{\beta}}) = \arg\max_{\boldsymbol{\alpha},\boldsymbol{\beta}} \pi(\boldsymbol{\alpha}, \boldsymbol{\beta}|\mathbf{y}, \boldsymbol{\gamma})$ and whose covariance matrix is equal to the the inverse Hessian of the conditional log-density $\log \pi(\boldsymbol{\alpha}, \boldsymbol{\beta}|\mathbf{y}, \boldsymbol{\gamma})$ evaluated at the conditional MAP $(\hat{\boldsymbol{\alpha}}, \hat{\boldsymbol{\beta}})$. The expression on the right-hand side of (S3) is

evaluated at $(\hat{\boldsymbol{\alpha}}, \hat{\boldsymbol{\beta}})$. Computing the MAP and the relevant Hessian is straightforward using standard optimizers. Note further that the density in the numerator of (S3), $\pi(\boldsymbol{\gamma}, \boldsymbol{\alpha}, \boldsymbol{\beta}|\mathbf{y})$ can be computed up to normalizing constant (which does not depend on $\boldsymbol{\gamma}$ or $\boldsymbol{\alpha}, \boldsymbol{\beta}$) as $\pi(\mathbf{y}|\boldsymbol{\alpha}, \boldsymbol{\beta})\pi(\boldsymbol{\alpha}, \boldsymbol{\beta}|\boldsymbol{\gamma})\pi(\boldsymbol{\gamma})$.

The approximate particle optimization algorithm is extremely similar to the one described in the main text. Namely, we sequentially update each particle in the particle set $\boldsymbol{\Gamma}$. To update a single particle, we sequentially update $\gamma^{(\alpha)}$ and $\gamma^{(\beta)}$ using the same suite of coarse and fine transitions. To update σ^2 we maximize the conditional posterior $\pi(\sigma^2|\hat{\alpha}, \hat{\beta}, \gamma^{(\alpha)}, \gamma^{(\beta)})$, where $\hat{\alpha}, \hat{\beta}$ is the conditional MAP corresponding to $\boldsymbol{\gamma}$. In these updates, we approximate $\pi(\boldsymbol{\gamma}|\mathbf{y})$ for every candidate particle $(\gamma^{(\alpha)}, \gamma^{(\beta)}, \sigma^2)$ that we consider.

S6.1 Illustration on synthetic data

To understand the performance of our approximate `PartOpt`, we performed a simulation study very similar to that described in Section S3.1. Specifically, we generated observations $c_{it} \sim \text{Pois}(\exp\{\alpha_i + \beta_i x_t\})$ where the α_i's and β_i's were drawn from a CAR-within-cluster distribution. We considered three settings of the α_i's and β_i's, one where the grand cluster means were highly separated (first row of Figure S12), one where the grand cluster means were moderately separated (second row of Figure S12), and one where the grand cluster means were very close in value (third row of Figure S12). We ran our approximate `PartOpt` with $L = 10$ particles and penalties $\lambda = 1, 10$, and 100.

For all values of λ, the particle set contained the true partitions $\gamma^{(\alpha)}$ and $\gamma^{(\beta)}$ shown in Figure S12. Like the simulations reported in the main manuscript for the Gaussian regression setting, we found that when $\lambda = 1$, many particles collapsed to the same point. However, we recovered a much more diverse particle set when we set $\lambda = 10$ and $\lambda = 100$. Figure S13 shows the $L = 10$ particles recovered when we ran the approximate `PartOpt` on data generated from (S2) with the α_i's and β_i's from the high separation setting, shown in the top panel Figure S12. Similarly, Figure S14 shows the recovered particles for the moderate separation setting, when $\lambda = 10$. Finally, Figure S15 shows the recovered particles for the low separation setting, when $\lambda = 10$. In the high and moderate cluster separation settings, all of the estimated partitions are extremely close to the true partitions and the corresponding conditional posterior means of α_i and β_i are quite close to the true values. In the low separation setting, the particle set recovered partitions quite different from the ones used to generate the data. The behavior of the approximate `PartOpt` in the low separation

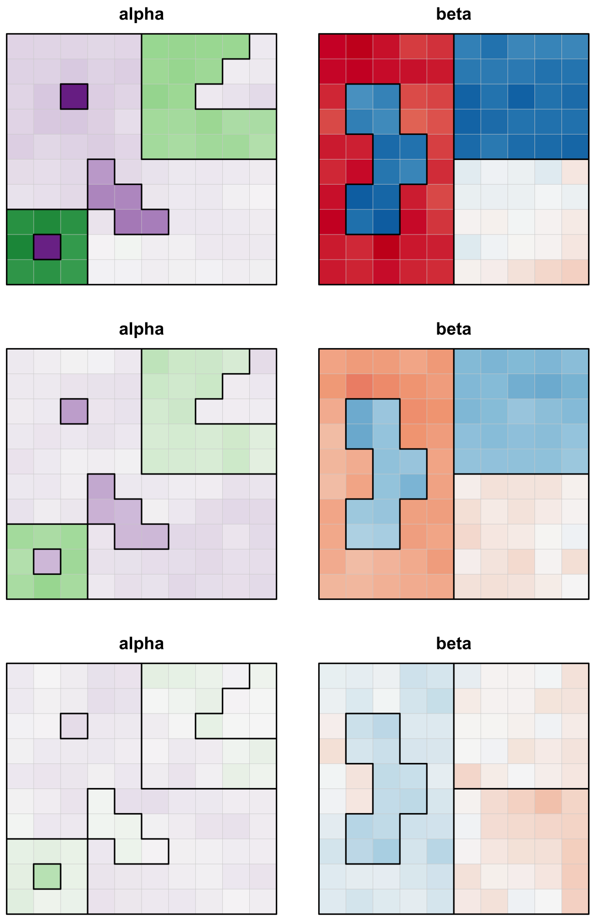

Figure S12: The true partition $\gamma^{(\alpha)}$ and $\gamma^{(\beta)}$ used to generate the synthetic data to test the extension of Particle Optimization to non-conjugate models. First row: high cluster separation configuration. Second row: moderate cluster separation configuration. Third row: low cluster separation configuration.

setting is not surprising: when there is little between-cluster variation in parameter values, the posterior strongly favors a single large cluster instead of several smaller clusters that all containing similar parameter values.

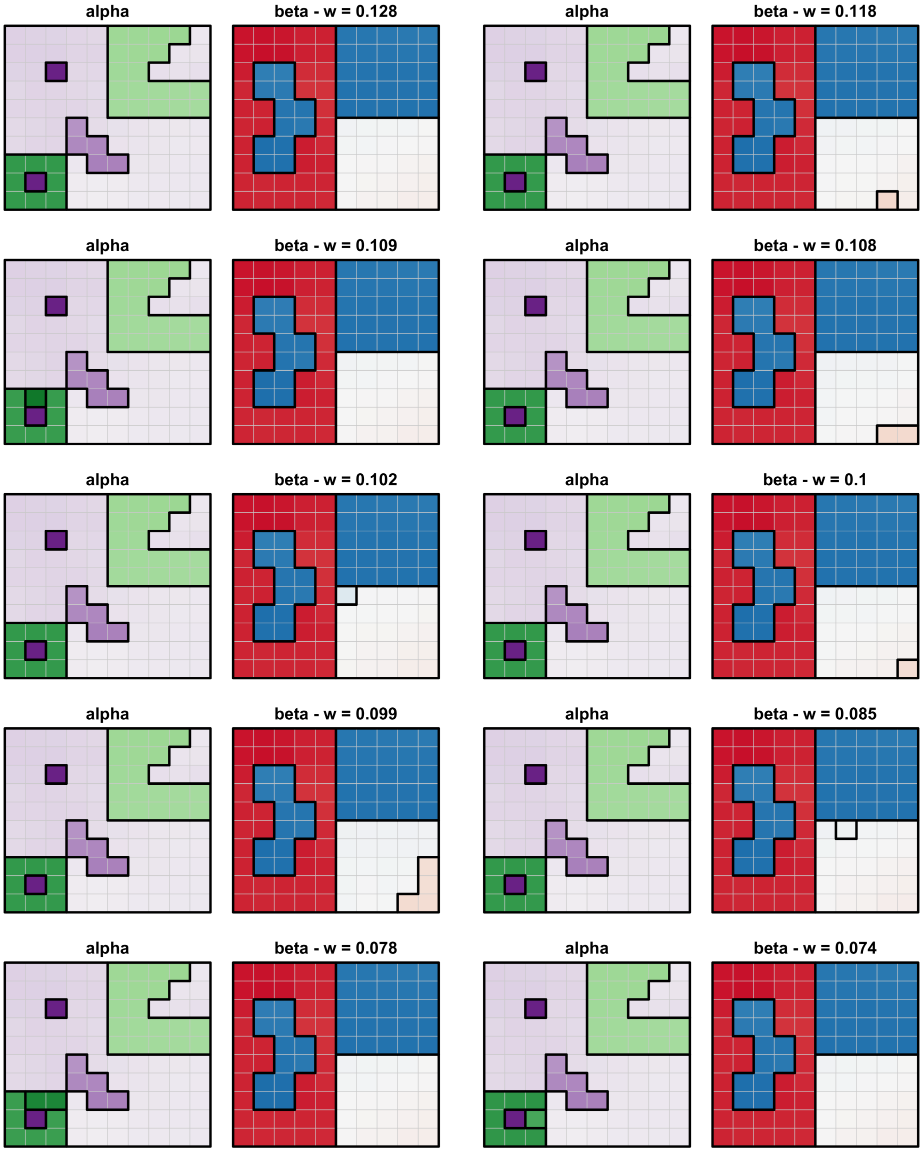

Figure S13: The estimate particles $(\gamma^{(\alpha)}, \gamma^{(\beta)})$ recovered for $\lambda = 10$ in the high separation setting, and the weight associated to each one.

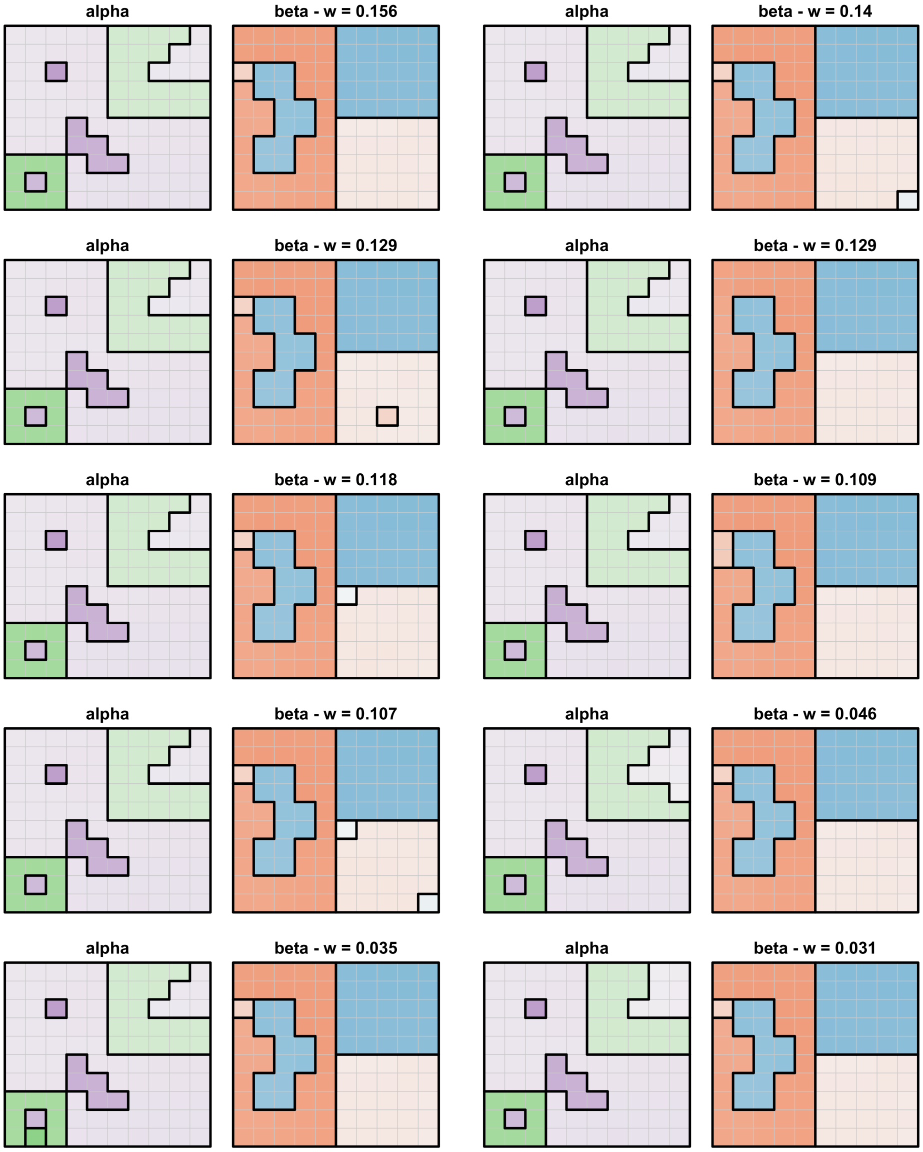

Figure S14: The estimate particles $(\gamma^{(\alpha)}, \gamma^{(\beta)})$ recovered for $\lambda = 10$ in the moderate separation setting, and the weight associated to each one.

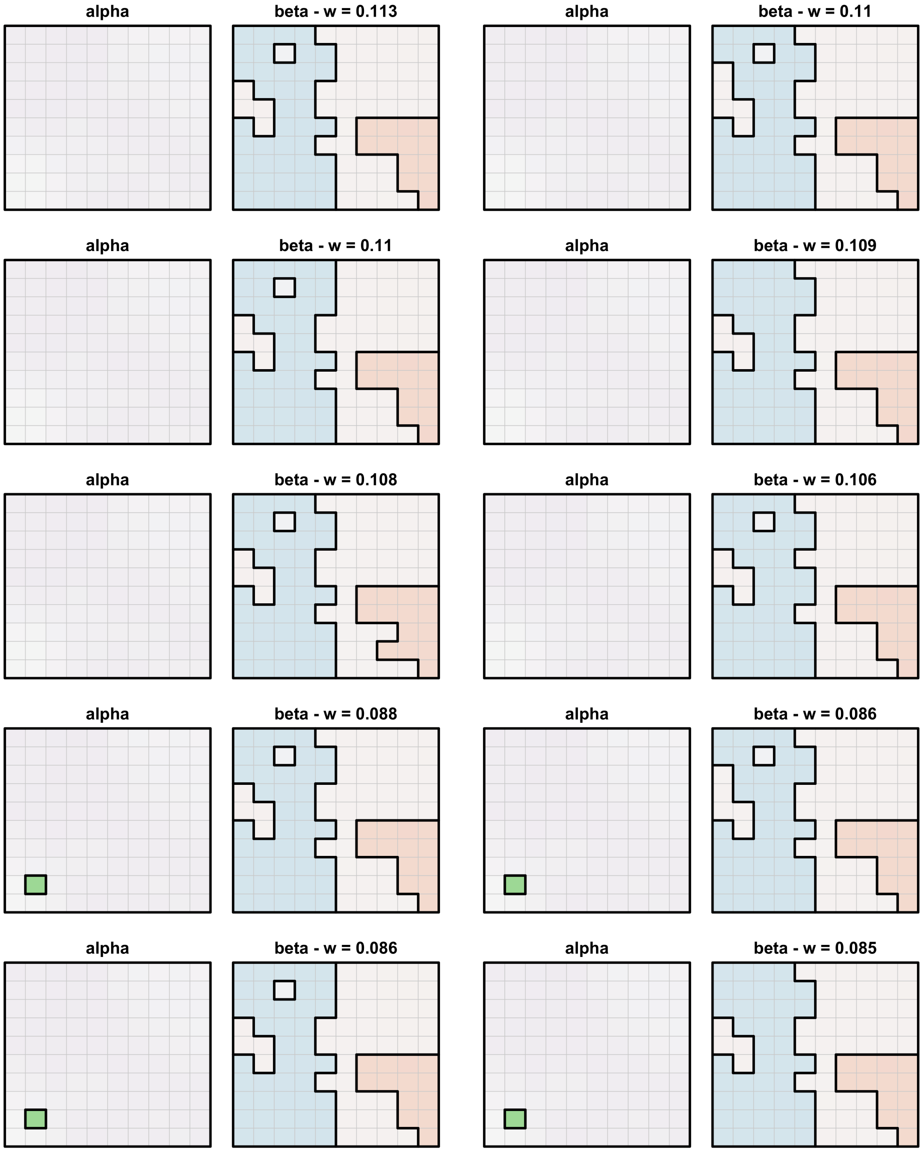

Figure S15: The estimate particles $(\gamma^{(\alpha)}, \gamma^{(\beta)})$ recovered for $\lambda = 10$ in the low separation setting, and the weight associated to each one.

References

Gelfand, A. E. and Vounatsou, P. (2003). Proper multivariate conditional autoregressive models for spatial data analysis. *Biostatistics*, 4(1):11–25.
